# ERRα negatively regulates type I interferon induction by inhibiting TBK1-IRF3 interaction

**DOI:** 10.1371/journal.ppat.1006347

**Published:** 2017-06-07

**Authors:** Xiang He, Shengli Ma, Yinyin Tian, Congwen Wei, Yongjie Zhu, Feng Li, Pingping Zhang, Penghao Wang, Yanhong Zhang, Hui Zhong

**Affiliations:** 1 State Key Laboratory of Pathogen and Biosecurity, Beijing Institute of Biotechnology, Beijing, P.R. China; 2 Institute of Healthy Science, Anhui University, Hefei, Anhui, P.R. China; Georgia State University, UNITED STATES

## Abstract

Estrogen-related receptor α (ERRα) is a member of the nuclear receptor superfamily controlling energy homeostasis; however, its precise role in regulating antiviral innate immunity remains to be clarified. Here, we showed that ERRα deficiency conferred resistance to viral infection both *in vivo* and *in vitro*. Mechanistically, ERRα inhibited the production of type-I interferon (IFN-I) and the expression of multiple interferon-stimulated genes (ISGs). Furthermore, we found that viral infection induced TBK1-dependent ERRα stabilization, which in turn associated with TBK1 and IRF3 to impede the formation of TBK1-IRF3, IRF3 phosphorylation, IRF3 dimerization, and the DNA binding affinity of IRF3. The effect of ERRα on IFN-I production was independent of its transcriptional activity and PCG-1α. Notably, ERRα chemical inhibitor XCT790 has broad antiviral potency. This work not only identifies ERRα as a critical negative regulator of antiviral signaling, but also provides a potential target for future antiviral therapy.

## Author summary

As a member of the nuclear receptor superfamily involved in metabolism signaling, the precise role of ERRα in antiviral innate immunity remains to be clarified. Here, we showed that ERRα deficiency led to increased interferon production, resulting in enhanced resistance to viral infection both *in vivo* and *in vitro*. Mechanistically, viral infection induced TBK1-dependent ERRα stabilization, which in turn increased its binding to TBK1 and IRF3 to prevent the formation of a functional TBK1-IRF3 complex. The effect of ERRα on IFN-I production was independent of its transcriptional activity and PCG-1α. Notably, ERRα chemical inhibitor XCT790 has broad antiviral potency. Taken together, our results identified ERRα as an important negative downstream regulator of TBK1 in RLRs-TLRs signaling pathways and suggested a potential therapeutic target for viral infection.

## Introduction

The innate immune system plays important roles in the detection and elimination of invading pathogens. The host senses viral and bacterial pathogen invasion via the recognition of pathogen-associated molecular patterns (PAMPs) by pattern recognition receptors (PRRs), including membrane-bound Toll-like receptors (TLRs) and cytosolic sensory molecules, such as RIG-like receptors (RLRs) and NOD-like receptors (NLRs). These then activate a series of signal cascades, leading to the production of IFN-I and proinflammatory cytokines. Upon viral infection, TLRs detect pathogen nucleic acids in the lumen of endosomes, whereas RLRs, DAI, IFI16, LRRFIP1 and cGAS sense pathogen nucleic acids in the cytoplasm [1–5]. TLRs-mediated signaling pathways associate with the adaptor protein MyD88 and TRIF, while RLRs recruit MAVS and STING. Both pathways ultimately converge on the activation of TBK1 upon adaptor recruitment. Activated TBK1 then phosphorylates IRF3, IRF5 and IRF7, triggers their dimerization and nuclear translocation, and activates IFN-I expression. Secreted IFN-α/β further activates downstream signaling pathways to induce a wide range of antiviral genes and elicit cellular antiviral responses.

As a critical kinase involved in antiviral immunity, TBK1 activity must be tightly regulated to maintain immune homeostasis. Various mechanisms have been reported to positively or negatively regulate IFN-I production through effects on TBK1. Nrdp1 [6] and GSK-β [7] enhance TBK1 activity by catalyzing Lys63-linked polyubiquitination or promoting TBK1 self-activation, respectively. TAX1BP1, A20 and NLRP4 terminate antiviral signaling by promoting TBK1 degradation or disrupting the Lys63-linked polyubiquitination of TBK1 [8,9]. Affecting the formation of functional TBK1-containing complexes is another major mechanism that modulates antiviral immune responses. For example, HSP90 facilitates TBK1-IRF3 complex formation through TBK1 stabilization [10]. MIP-T3 and SIKE negatively regulate IFN-β production by inhibiting the formation of TRAF3-TBK1 and TBK1-IRF3 complexes [11,12].

ERRα is an orphan receptor that shares high sequence identity with nuclear receptors α/β (ERα and ERβ). Nevertheless, a functional analysis has indicated that the majority of genes regulated by ERRα are distinct from those controlled by ERα [13]. ERRα possesses a central zinc finger DNA binding domain (DBD), a conserved C-terminal domain with a putative ligand binding domain (LBD) and a less conserved N-terminal region [14]. Although the natural ligand of ERRα is unknown, ERRα activates the transcription of genes that are involved in mitochondrial function and energy metabolism [15–23]. However, the roles of ERRα may not be limited to the direct transcriptional regulation of metabolism. For instance, ERRα induces orientated cellular migration by promoting the transcriptional expression of TNFAIP1, which subsequently destabilizes RHOA [24]. Under hypoxic conditions, ERRα acts as a co-activator to enhance HIF-mediated hypoxic responses by associating with HIF1α [25]. Mice lacking ERRα produce fewer reactive oxygen species (ROS) in macrophages and are susceptible to *Listeria monocytogenes* (LM) infection in response to IFN-γ treatment [22]. A recent study showed that ERRα negatively regulates TLR-induced inflammation by promoting the expression of A20 [26]. Hwang and colleagues proposed that ERRα is important for providing a favorable metabolic environment that supports optimal cytomegalovirus replication [27].

In the present study, we found that the inhibition of ERRα yielded broad anti-viral activities. ERRα deficiency induced significantly higher levels of IFN-β and increased the expression of a panel of ISGs in response to viral infection. Mechanistically, viral infection stabilized ERRα expression, which in turn associated with TBK1 to impede the formation of the TBK1-IRF3 complex, IRF3 phosphorylation, IRF3 dimerization and the DNA binding affinity of IRF3. Therefore, ERRα is a feedback inhibitor of antiviral innate immunity.

## Results

### ERRα deficiency confers resistance to viral infection both *in vivo* and *in vitro*

**Fig 1:**
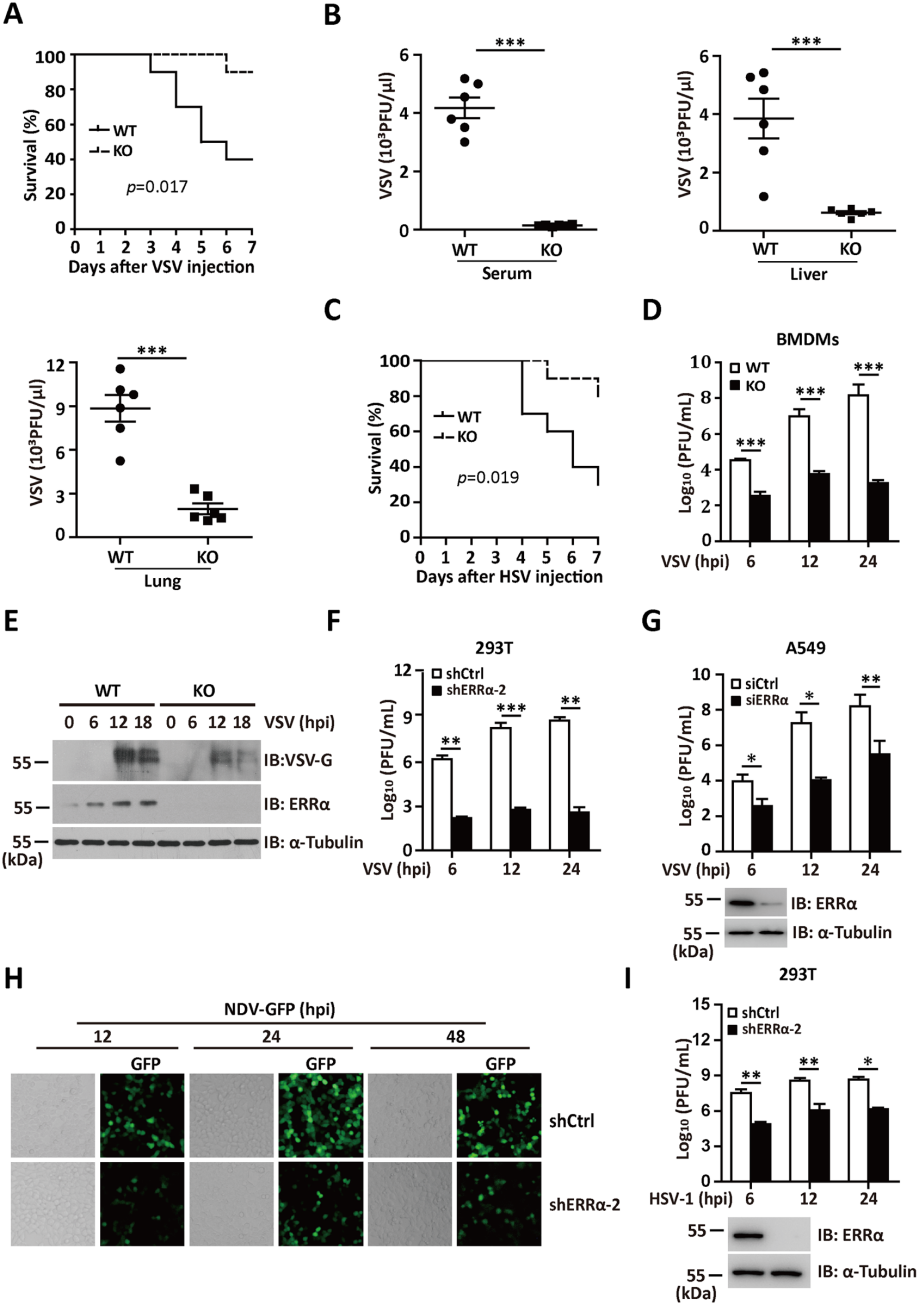
ERRα deficiency confers resistance to viral infection both *in vivo* and *in vitro*. (A) Survival of 6-week-old WT and ERRα-KO mice given tail vein injections of VSV (1 x 10^8^ pfu/g) (n = 10 per group). (B) Plaque assay of VSV loads in sera, liver or lung isolated from WT and ERRα-KO mice given tail vein injections of VSV for 24 h (n = 6 per group). (C) Survival of 6-week-old WT and ERRα-KO mice given tail vein injections of HSV-1 (1 x 10^7^ pfu/g) (n = 10 per group). (D) Plaque assay of VSV loads in supernatants of WT and ERRα-KO BMDMs infected with VSV (MOI = 1.0) for the indicated times. (E) Immunoblotting analysis of ERRα and VSV-G protein levels in lysates of WT and ERRα-KO BMDMs infected with VSV (MOI = 1.0) for the indicated times. (F) Plaque assay of VSV loads in supernatants from control (shCtrl) or ERRα knockdown (shERRα-2) 293T cells followed by VSV (MOI = 1.0) infection for the indicated times. (G) Plaque assay of VSV loads in supernatants from control (siCtrl) or ERRα knockdown (siERRα) A549 cells followed by VSV (MOI = 1.0) infection for the indicated times. (H) Microscopy imaging of shCtrl or shERRα-2 293T cells infected with NDV-GFP (MOI = 1.0) for the indicated times. (I) Plaque assay of HSV-1 loads in supernatants of shCtrl or shERRα-2 293T cells infected with VSV (MOI = 1.0) for the indicated times. Loading controls were shown in the lower panel of some Figures. Cell-based studies were performed independently at least three times with comparable results. The data are presented as the means ± SEM.

An increasing amount of evidence has demonstrated the crosstalk between the innate immune response and metabolic pathways; however, the precise molecule that links the two systems remains to be clarified. As a member of the nuclear receptor superfamily involved in metabolic signaling, the precise role of ERRα in regulating antiviral innate immunity remains to be clarified. To evaluate the importance of ERRα in viral infection, we first infected wild type (WT) and ERRα-KO (ERRα-KO) mice with vesicular stomatitis virus (VSV). As shown in Fig 1A, the ERRα-KO mice were more resistant to VSV infection in the overall survival assay. VSV titers in sera, liver and lung isolated from ERRα-KO mice were also significantly reduced, compared to WT mice on day 3 post-infection (Fig 1B). We next infected WT type and ERRα-KO mice with herpes simplex virus type 1 (HSV-1), a DNA virus. As shown in Fig 1C, ERRα-KO mice were more resistant to lethal HSV-1 infection.

To further determine the role of ERRα in viral infection, we examined the effects of ERRα deficiency on the replication of various viruses in isolated and cultured cells. Bone marrow-derived macrophages (BMDMs) from the ERRα-KO mice showed lower VSV production than the cells from the WT mice did (Fig 1D and 1E and S1A Fig). Moreover, stable ERRα knockdown clone 2 (shERRα-2) in human 293T (S1B Fig) resulted in decreased VSV titers (Fig 1F) and enhanced cell viability in response to VSV infection (S1C Fig). Similarly, the expression of siRNA for ERRα (siERRα) in A549 cells also resulted in lower viral titers in the supernatant than transfection with control siRNA (siCtrl; Fig 1G). Conversely, 293T cells with overexpressed ERRα showed significantly increased VSV titers in the supernatant (S1D Fig). We next infected 293T cells with GFP-tagged Newcastle disease virus (NDV-GFP) and HSV. Based on quantification by fluorescence microscopy, flow cytometry and plaque assays, both NDV-GFP replication (Fig 1H and S1E and S1F Fig) and HSV-1 production (Fig 1I) was greatly reduced in the shERRα-2 cells. These data collectively suggest that ERRα deficiency confers resistance to viral infections both *in vitro* and *in vivo*.

### ERRα knockdown increases the expression of multiple antiviral genes

**Fig 2:**
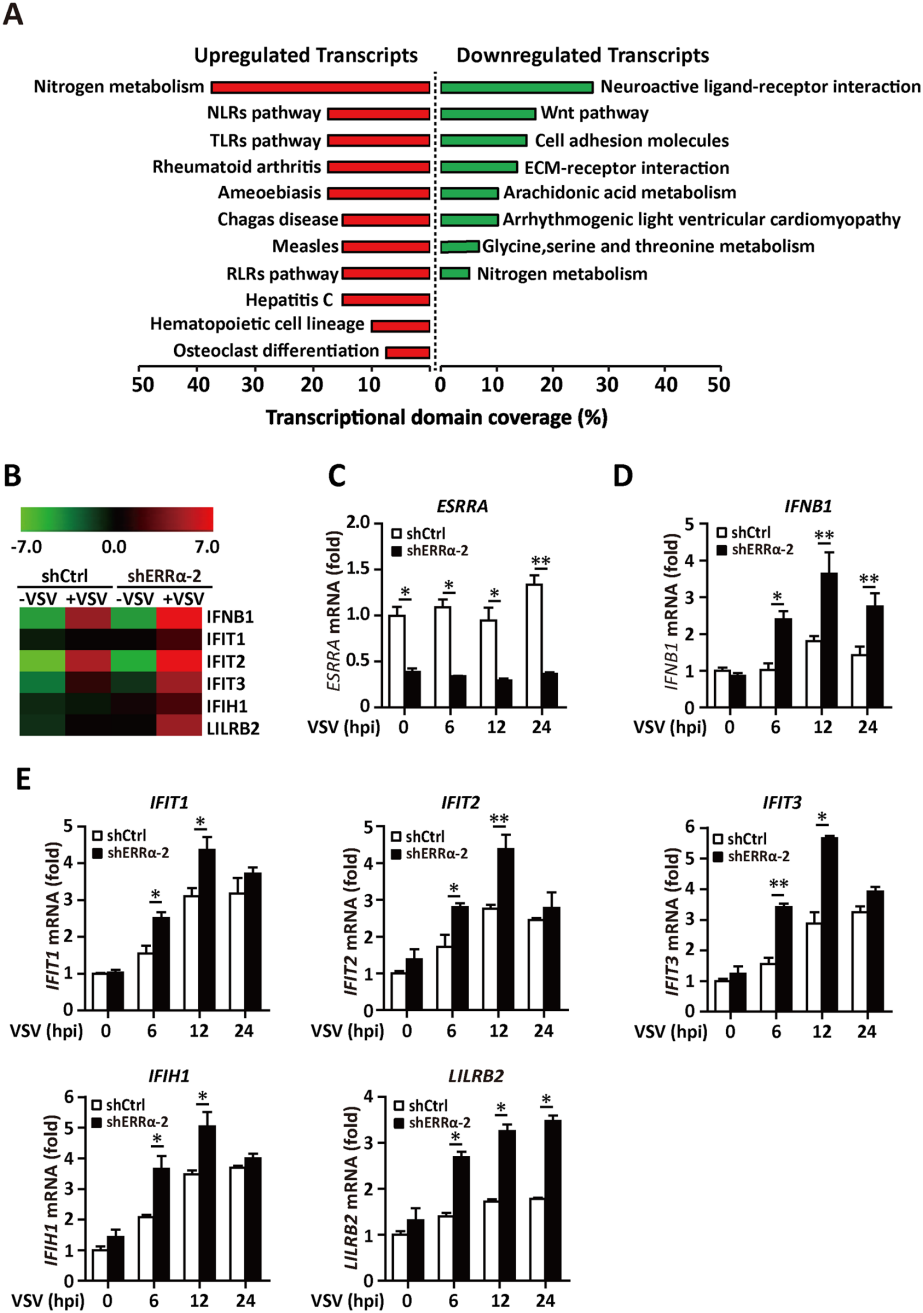
ERRα knockdown increases the expression of multiple antiviral genes. (A-B) Microarray analysis of shCtrl and shERRα-2 293T cells following VSV infection for 12 h. (A) Enrichment of KEGG pathways by FunNet analysis for genes upregulated or downregulated in shERRα-2-VSV cells relative to their expression in shCtrl-VSV cells (adjusted to FC ≥ 2) in at least one comparison. (B) Heatmap of upregulated IFN-I responsive genes. The color represents normalized expression of genes in shERRα-2 cells relative to their expression in shCtrl cells. (C-E) qPCR analysis of *ESRRA*, *IFNB1*, *IFIT1*, *IFIT2*, *IFIT3*, *IFIH1* and *LILRB2* mRNA expression in shCtrl and shERRα-2 293T cells infected with VSV for the indicated times. The data were normalized to the expression of the *β-Actin* reference gene. Cell-based studies were performed independently at least three times with comparable results. The data are presented as the means ± SEM.

A recent study reported that ERRα was required for the efficient production of cytomegalovirus progeny by providing a favorable metabolic environment. Here, we used microarray analysis to determine the expression of genes altered by ERRα inhibition. ShCtrl and shERRα-2 clones in the 293T cell line (S2A Fig) were analyzed by microarray assay 12 h after VSV infection. First, we subjected genes that exhibited 1.5-fold changes to FunNet analysis to determine the several top pathways regulated by ERRα upon viral infection. Based on this analysis, the top eight most significant downregulated KEGG pathways following ERRα inhibition were associated with metabolic pathways, Wnt signaling, and adherens junctions, which have been reported previously [28–30] (Fig 2A). Interestingly, the NLRs, TLRs and RLRs innate immune pathways were ranked as the top upregulated signaling pathways (Fig 2A). Specially, *IFNB1* and several interferon-responsive genes, including *IFIT1*, *IFIT2*, *IFIT3*, *IFIH1* and *LILRB2*, were induced at greater levels in infected cells in which ERRα was knocked down (Fig 2B). The increased expression of IFN-β and responsive genes by ERRα knockdown was validated by quantitative real-time PCR (qRT-PCR) on RNA samples prepared at various time points after VSV infection (Fig 2C–2E). Consistent with a previous report, RNA corresponding to genes that encode triacylglycerol metabolism and glycolytic proteins were downregulated by at least a factor of two following knockdown of ERRα, including *CRAT*, *ACO2*, *LIPE*, and *BDH1* (S2B Fig) [22,24,31,32].

### ERRα negatively regulates IFN-I production both *in vitro* and *in vivo*

**Fig 3:**
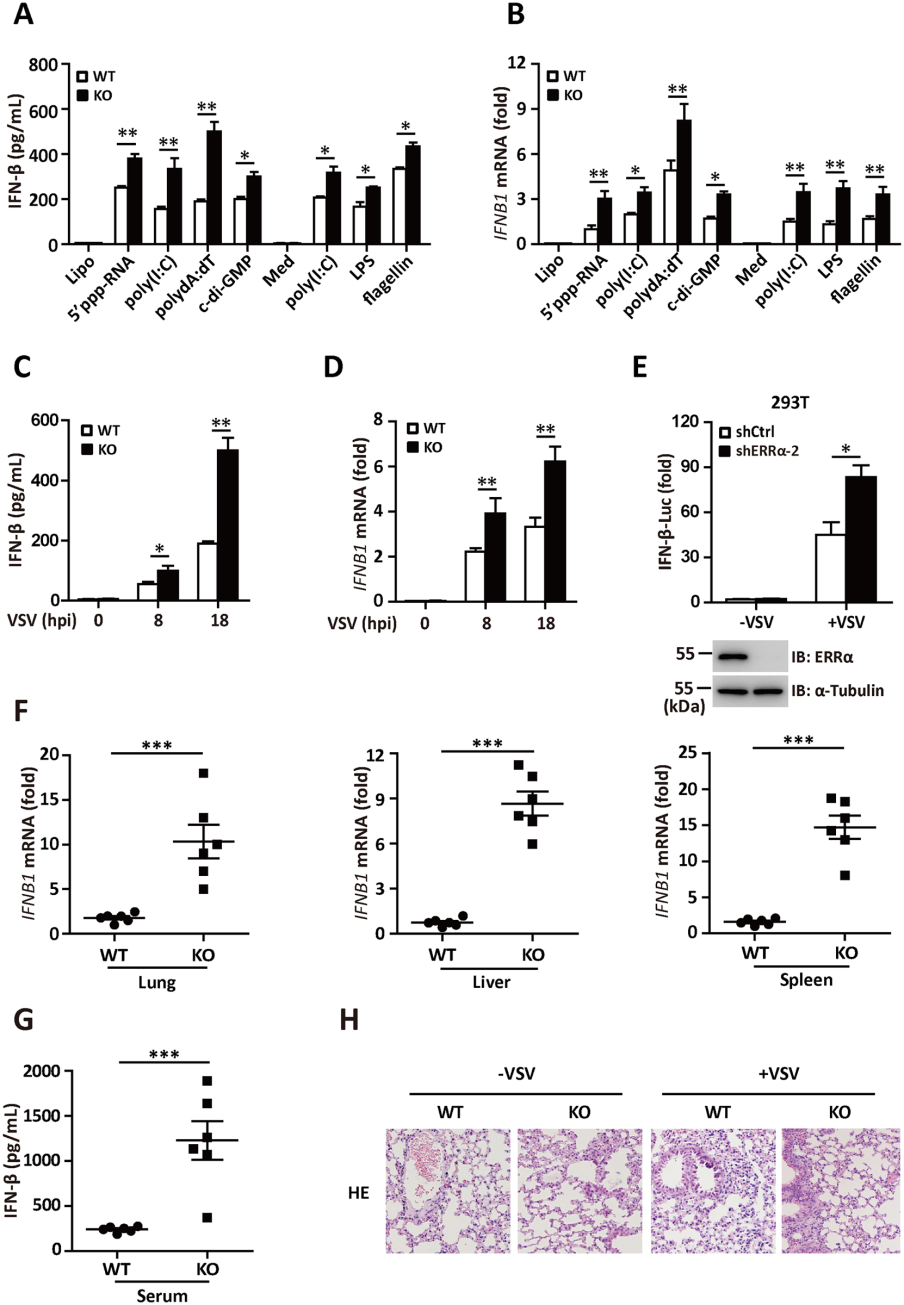
ERRα negatively regulates IFN-I production both *in vitro* and *in vivo*. (A) ELISA analysis of IFN-β secretion in WT and ERRα-KO BMDMs transfected with Lipofectamine, 5’-ppp dsRNA (0.1 g/ml), poly (I:C) (0.1 g /ml), poly(dA:dT) (0.1 g /ml) and c-di-GMP (10 g /ml) or incubated with the Medium control (Med), HMW poly(I:C) (0.5 g/ml), LPS (0.1 g /ml) or flagellin (1 g /ml) for 12 h. (B) qRT-PCR analysis of IFN-β mRNA expression in WT and ERRα-KO BMDMs treated as shown in Fig 3A. (C-D) ELISA analysis of IFN-β (C) and qRT-PCR analysis of IFN-β mRNA (D) expression in WT and ERRα-KO BMDMs infected with VSV for the indicated hours. (E) IFN-β promoter luciferase activity assays in shCtrl or shERRα-2 293T cell lines infected with VSV (MOI = 1.0) for 12 h. (F) qRT-PCR analysis of IFN-β mRNA expression in lung, liver or spleen isolated from WT and ERRα-KO mice given tail vein injections of VSV for 24 h (n = 6 per group). (G) ELISA analysis of IFN-β protein in sera from WT and ERRα-KO mice given tail vein injections of VSV for 24 h (n = 6 per group). (H) Pathology of WT and ERRα-KO mice in response to VSV. Scale bar, 100 mm. HE staining of lung sections. Loading controls were shown in the lower panel of some Figures. Cell-based studies were performed independently at least three times with comparable results. The data are presented as the means ± SEM.

To further investigate the role of ERRα in innate immunity, we isolated primary BMDMs from WT or ERRα-KO mice and measured the expression of IFN-β expression in response to RLR-, cGAS- and DDX41-activating stimuli. We transfected 5’-triphosphate(5’-ppp) dsRNA, poly(I:C), poly(dA:dT), and cyclic diguanosine monophosphate(c-di-GMP) in the WT and ERRα-KO BMDMs. IFN-β secretion was significantly increased in the ERRα-KO BMDMs (Fig 3A). BMDMs from the ERRα-KO mice also produced significantly more IFN-β in response to poly(I:C), lipopolysaccharide (LPS) or flagellin incubation, which activate TLR3, TLR4 or TLR5, respectively (Fig 3A). Consistent with this result, ERRα-KO BMDMs showed upregulated *IFNB1* mRNA expression in response to all the agonists tested (Fig 3B). VSV is a negative-strand ssRNA rhabdovirus that activates IFN-α/β through RIG-I [33]. VSV-induced IFN-β secretion and *IFNB1* mRNA expression was also greatly enhanced in ERRα-KO macrophages in a time-dependent manner (Fig 3C and 3D). Therefore, ERRα is involved in negative regulation of the RLRs, DDX41 and TLRs signaling pathways. In reporter assays, shERRα-2 293T cells (Fig 3E) and siERRα A549 cells (S3A Fig) dramatically potentiated VSV-induced activation of the IFN-β promoter.

To further determine the role of ERRα in type I interferon induction *in vivo*, we infected WT and ERRα-KO mice with VSV. In keeping with our *in vitro* data, the induction of *IFNB1* mRNA expression was greatly enhanced in the organs of ERRα-KO mice compared to WT mice infected with VSV (Fig 3F). Furthermore, we detected more circulating IFN-β in the blood of ERRα-KO mice on day 3 after VSV infection (Fig 3G). The lungs of ERRα-KO mice demonstrated significantly less inflammation, with reduced epithelial damage, mononuclear cell infiltrates and alveolitis (Fig 3H). Thus, ERRα functions as a negative regulator of type I interferon production upon viral infection.

### ERRα associates with TBK1, IKKε and IRF3

**Fig 4:**
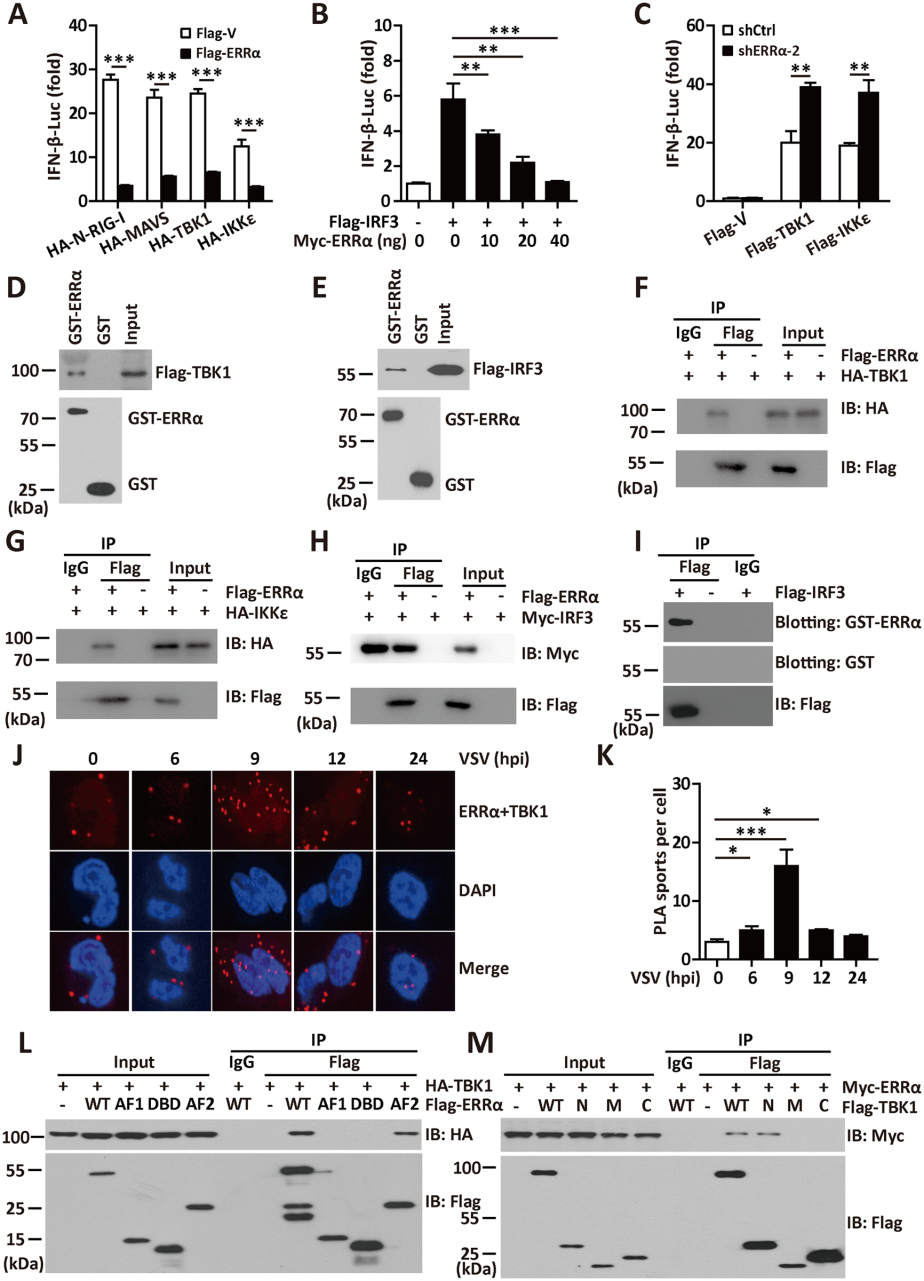
ERRα associates with TBK1, IKKε and IRF3. (A-B) IFN-β promoter luciferase activity assay in 293T cells transfected with the indicated plasmids. (C) IFN-β promoter luciferase activity assays in shCtrl or shERRα-2 cells transfected with the indicated plasmids. (D-E) GST pulldown assay in 293T cells transfected with Flag-TBK1 (D) or Flag-IRF3 (E). (F-H) Immunoprecipitation analysis in 293T cells transfected with Flag-ERRα and HA-TBK1 (F), HA-IKKε (G) or Myc-IRF3 (H). (I) Far-western analysis in 293T cells transfected with Flag-IRF3. *In situ* PLA assay of the ERRα-TBK1 complex in 293T cells infected with VSV (MOI = 1.0) for the indicated times. ERRα-TBK1 complex, red; nuclei, blue. Quantification of PLA signals per cell in Fig 4J presented relative to control cells treated with solvent. (L-M) Immunoprecipitation analysis in 293T cells transfected with the indicated plasmids. Cell-based studies were performed independently at least three times with comparable results. The data are presented as the means ± SEM.

Various activators, such as RIG-I, MAVS, TBK1, and IKKε, have been reported to be involved in the virus-triggered IRF3 activation pathway [34]. Overexpressed ERRα inhibited IFN-β (Fig 4A and S4A Fig), IRF3 (S4B Fig) and ISRE activation (S4C Fig) induced by these activators in a luciferase reporter assay. Overexpression of IRF3 in 293T cells potently activated the IFN-β and ISRE promoters, while as little as 0.01 μg of ERRα was sufficient to cause potent repression (>80%) of IFN-β (Fig 4B and S4D Fig) and ISRE (S4E Fig). The extent of the suppression increased with increasing amounts of ERRα, suggesting that ERRα inhibited the induction of IFN-β by IRF3 in a dose-dependent manner. The phosphorylation, dimerization and nuclear translocation of IRF3 necessary for the activation of *IFNB1* transcription require IKKε and TBK1. Knockdown of ERRα expression significantly enhanced IFN-β promoter activation by TBK1 or IKKε (Fig 4C and S4F Fig).

Our observation that ERRα inhibited the IFN-I production by targeting TBK1 and IRF3 raised the possibility that ERRα might physically interact with these targets. To test this possibility, lysates with ectopic expression of TBK1 or IRF3 from 293T cells were incubated with GST or the GST-ERRα fusion protein. Both TBK1 and IRF3 could bind to GST-ERRα but not to GST (Fig 4D and 4E), demonstrating an *in vitro* interaction of ERRα with TBK1 and IRF3. To test whether ERRα bound to TBK1 and IRF3 in mammalian cells, Flag-ERRα was transfected together with HA-TBK1, HA-IRF3 or HA-IKKε. Immunoblotting analysis of anti-Flag immunoprecipitate with an anti-HA antibody showed a significant association between Flag-ERRα and HA-TBK1, HA-IKKε and HA-IRF3 (Fig 4F–4H). A far-western assay also revealed a direct interaction between ERRα and IRF3 (Fig 4I). Importantly, we visualized endogenous ERRα-TBK1 complex formation using an *in situ* proximity ligation assay (PLA). We observed few spots representing the ERRα-TBK1 complex in uninfected 293T cells, while the spots increased significantly at 9 hpi and began to reduce at 12 hpi (Fig 4J and 4K). A domain mapping experiment indicated that the N-terminal domain of TBK1 [35] (amino acids 1–510) was required for its interaction with the AF2 domain of ERRα (Fig 4L and 4M). These data suggest that the interaction between TBK1, IRF3 and ERRα is responsible for the ERRα-mediated inhibition of antiviral signaling.

### ERRα regulates TBK1-IRF3 complex formation

**Fig 5:**
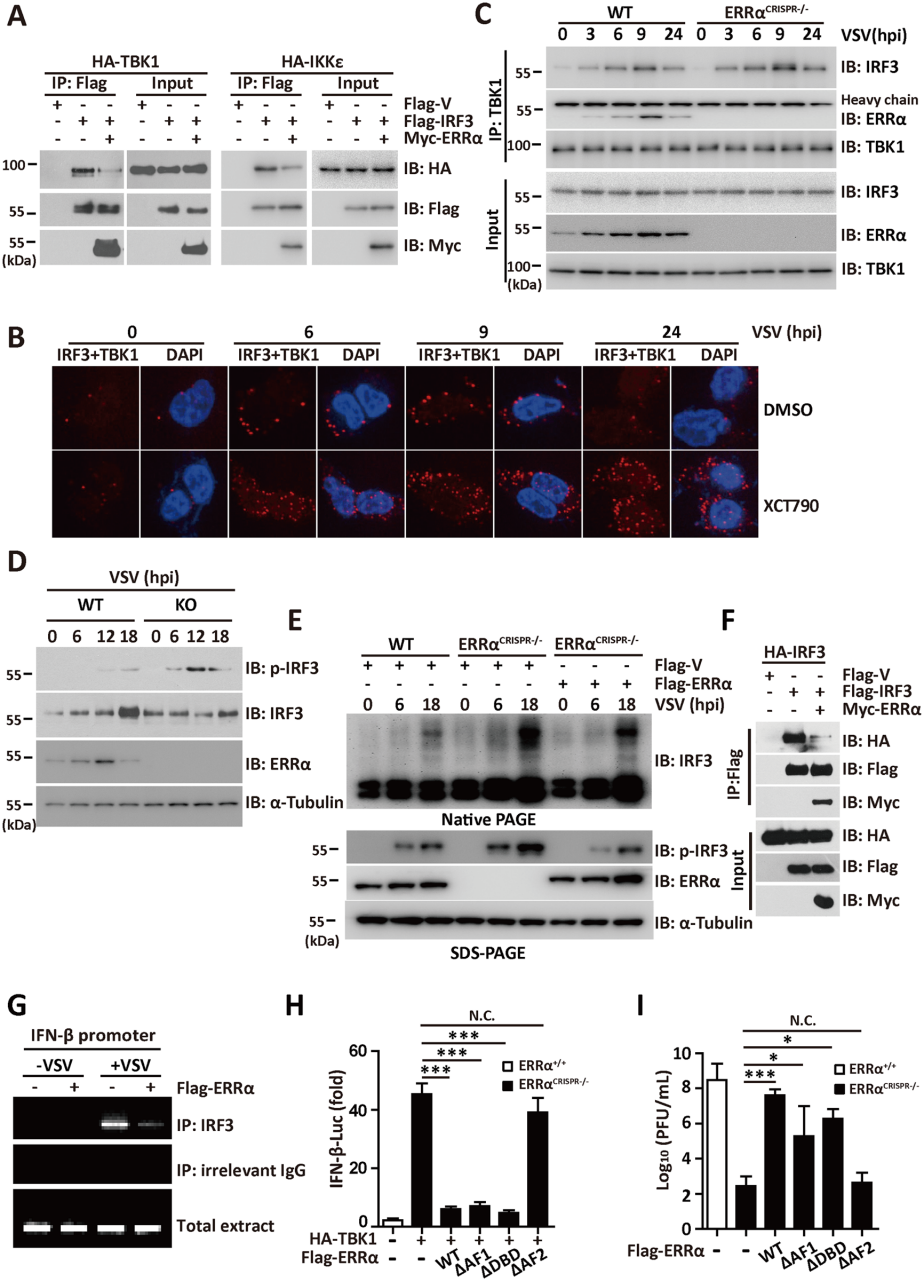
ERRα regulates TBK1-IRF3 complex formation. (A) Immunoprecipitation analysis in 293T cells transfected with Flag-IRF3 and HA-TBK1 or HA-IKKε in the presence or absence of Myc-ERRα. (B) *In situ* PLA assay of the IRF3-TBK1 complex in 293T cells infected with VSV (MOI = 1.0) in the presence or absence of 2.5 μM XCT790 for the indicated times. TBK1-IRF3 complex, red; nuclei, blue. (C) Immunoprecipitation analysis in WT and ERRα^CRISPR-/-^ cells infected with VSV (MOI = 1.0) for the indicated times. (D) Immunoblotting analysis in WT and ERRα-KO BMDMs infected with VSV (MOI = 1.0) for the indicated times. (E) Native gel electrophoresis (Native-PAGE) or SDS–PAGE anlaysis in WT and ERRα^CRISPR-/-^ cells transfected with the indicated expression plasmids and infected with VSV (MOI = 1.0) for the indicated times. (F) Immunoprecipitation analysis in 293T cells transfected with Flag-IRF3 and HA-IRF3 in the presence or absence of Myc-ERRα. ChIP assay in 293T cells transfected with Flag-ERRα and infected with VSV. PCR products were verified by agarose gel electrophoresis. (H) IFN-β promoter luciferase activity assay in ERRα^CRISPR-/-^ cells transfected with HA-TBK1 and Flag-ERRα or its mutants. (I) Plaque assay of VSV loads in supernatants of ERRα^CRISPR-/-^ cells transfected with Flag-ERRα or its mutants and infected with VSV (MOI = 1.0) for 12 h. Cell-based studies were performed independently at least three times with comparable results. The data are presented as the means ± SEM.

To further investigate the inhibitory mechanism underlying the role of ERRα in antiviral immune signaling, we explored the effect of ERRα on the TBK1-IRF3 interaction and IRF3 dimerization triggered by viral infection. Notably, the introduction of overexpressed ERRα destroyed the binding between TBK1, IKKε and IRF3 (Fig 5A). XCT790 is a specific inhibitor of ERRα, which reduces ERRα expression [36] (S5A Fig). By using *in situ* PLA, we found that the number of spots representing the IRF3-TBK1 complex induced by stimulation with VSV was greatly increased in 293T cells treated with XCT790 (Fig 5B and S5B Fig). In addition, we generated 293T cells lacking ERRα using the CRISPR/Cas9 system (ERRα^CRISPR-/-^). As shown in Fig 5C, the TBK1-ERRα and TBK1-IRF3 interaction began to increase at 3 hpi. In the absence of ERRα, a significantly increased TBK1-IRF3 interaction was observed upon VSV infection (Fig 5C and S5C Fig).

Type I interferon gene transcription is mediated primarily through transcription factor IRF3, which is localized inside the cytoplasm of resting cells. Upon stimulation, IRF3 is activated by serine/threonine phosphorylation, which leads to dimerization, nuclear translocation and binding to recognition sequences in the promoters and enhancers of type I interferon genes. We next attempted to dissect the effect of ERRα on the activity of IRF3. Notably, the signals for VSV-induced IRF3 phosphorylation were significantly higher in BMDMs isolated from the ERRα-KO mice (Fig 5D). Because IRF3 phosphorylation promotes its dimerization, we measured the dimerization of IRF3 using native PAGE gels. Flag-V or Flag-ERRα plasmids were transfected into ERRα^CRISPR-/-^ cells. As expected, IRF3 dimerization and IRF3 phosphorylation in response to VSV infection occurred at much higher levels in the ERRα^CRISPR-/-^ cells than in the WT cells or in the ERRα^CRISPR-/-^ cells rescued with ERRα (Fig 5E). Moreover, IRF3 dimerization was disrupted with the addition of ERRα (Fig 5F). Because the IRF3 dimer binds more strongly to DNA than does the IRF3 monomer, the influence of ERRα on IRF3 binding to the IFN-β promoter was measured using a ChIP assay. As shown in Fig 5G, the increased binding to the IFN-β promoter region by VSV infection was significantly blocked by overexpressed ERRα.

We then wanted to investigate whether the direct binding of ERRα to the N-terminal kinase domain of TBK1 has any functional relevance in ERRα-mediated type-I IFN inhibition. Consistent with the binding result, the AF2 domain of ERRα was required for the inhibition of VSV and TBK1-induced activation of the IFN-β promoter (S5D Fig and Fig 5E). TBK1-induced ISRE activation was also inhibited by the AF2 domain (S5F Fig). To further validate the role of the AF2 domain in regulating the production of IFN-β, we transfected HA-TBK1 along with Flag-vector, Flag-ERRα or Flag-ERRα deletion mutants into ERRα^CRISPR-/-^ cells. TBK1-induced IFN-β activation was inhibited by WT ERRα, AF1 or DBD deletion mutant, but not by the AF2 deletion mutant (Fig 5H and S5G Fig). Consistently, viral growth in ERRα^CRISPR-/-^ cells transfected WT ERRα, AF1 or DBD deletion mutant was greater than that in ERRα^CRISPR-/-^ cells transfected with the AF2 deletion mutant (Fig 5I and S5H Fig). Based on these experiments, we concluded that the binding of ERRα inhibits antiviral signaling through direct physical binding with TBK1.

### The transcriptional activity of ERRα is dispensable for its role in antiviral signaling

**Fig 6:**
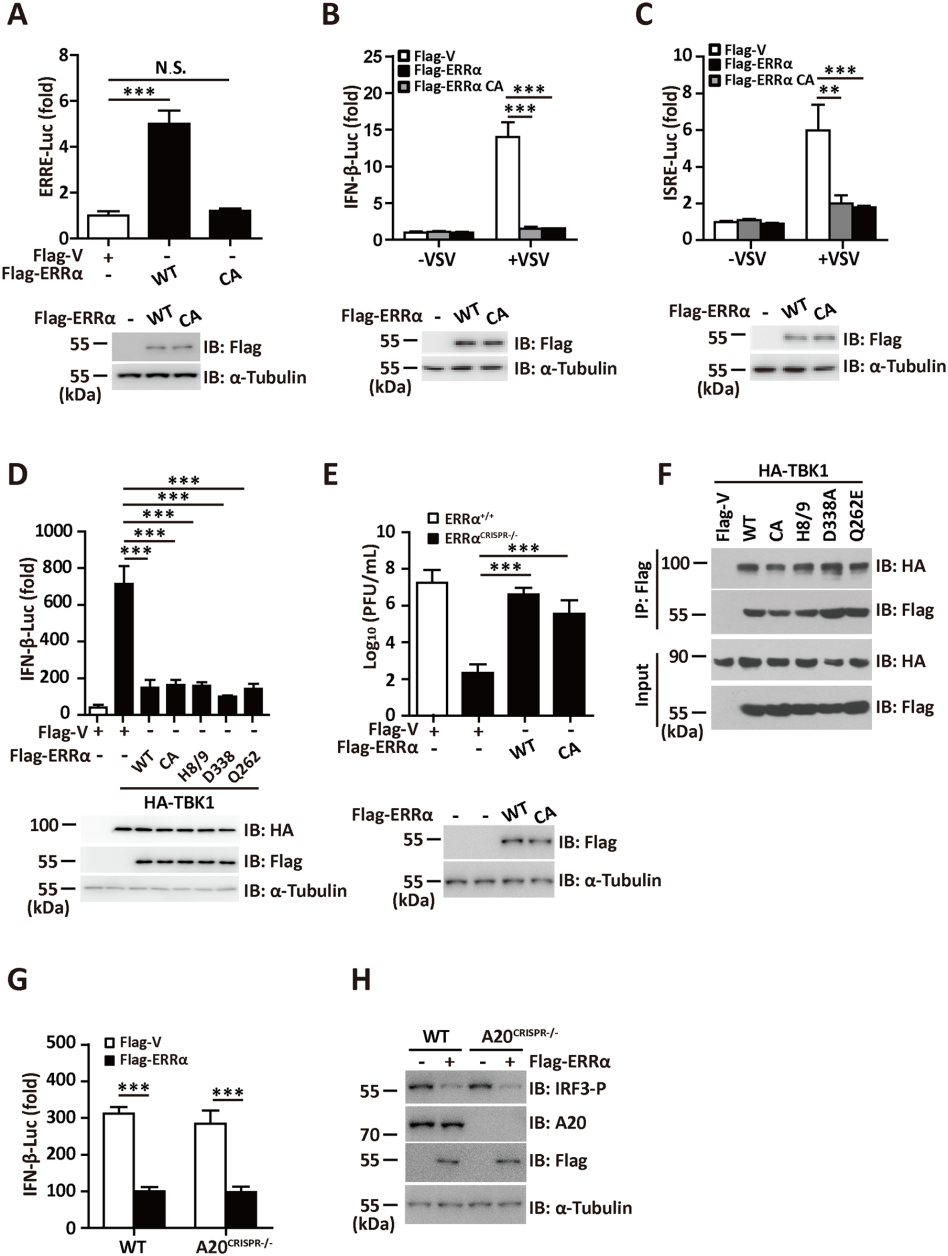
The transcriptional activity of ERRα is dispensable for its role in antiviral signaling. (A) ERRE promoter luciferase activity assay in 293T cells transfected with Flag-ERRα or Flag-ERRα CA mutant. (B-C) IFN-β (B) or ISRE promoter luciferase activity(C) assay in 293T cells transfected with Flag-ERRα or Flag-ERRα CA mutant and infected with VSV (MOI = 1.0) for 12 h. (D) IFN-β promoter luciferase activity assay in 293T cells transfected with HA-TBK1, Flag-ERRα or its mutants. (E) Plaque assay of VSV loads in supernatants from ERRα^CRISPR-/-^cells transfected with Flag-ERRα or Flag-ERRα CA mutant followed by VSV (MOI = 1.0) infection for the indicated times. (F) Immunoprecipitation analysis in 293T cells transfected with HA-TBK1 and Flag-ERRα or its mutants. (G) IFN-β promoter luciferase activity assay in WT and A20^CRISPR-/-^ cells transfected with Flag-ERRα. (H) Immunoblotting assay in WT and A20^CRISPR-/-^ cells transfected with Flag-ERRα. Loading controls were shown in the lower panel of some Figures. Cell-based studies were performed independently at least three times with comparable results. The data are presented as the means ± SEM.

Our results indicated that ERRα prevented the formation of functional TBK1-IRF3 complex and inhibited the binding affinity of IRF3 to impede IFN-α/β activation. However, whether the transcriptional activity of ERRα is required for the inhibition of this process is unknown. Similar to other nuclear receptors, the DBD domain of ERRα consist of two zinc-finger motifs: the first zinc-finger is responsible for the recognition of specific DNA binding sites, and the second zinc-finger mediates homo-dimerization of the nuclear receptors. Because cysteine residues in the zinc-finger motifs are critical for zinc ion binding, an ERRα CA mutant was constructed by changing the cysteines at positions 79, 96, 115, and 121 to alanines in order to abolish its transcriptional activity. We found that the ERRα CA mutant lost its ability to activate the ERRα promoters (Fig 6A); however, this mutant retained its ability to inhibit the activation of IFN-β and ISRE to levels as potent as the WT (Fig 6B and 6C). TBK1-induced IFN-β activation was equally inhibited by the wild type ERRα and CA mutant (Fig 6D). We then transfected Flag-ERRα or the Flag-ERRα CA mutant into ERRα^CRISPR-/-^ cells. Viral growth in ERRα^CRISPR-/-^ cells transfected with WT and CA mutant ERRα were greater than that in ERRα^CRISPR-/-^ control cells (Fig 6E).

PGC-1α usually acts as a transcriptional cofactor for ERRα in the regulation of metabolic signaling. A previous report revealed that substitution of the ERRα H8–H9 loop (amino acids 338–341, ERRα H8/9) with ERα amino acids 457–468 abolished its interaction with PGC-1α [37]. The ERRα point mutations D338A and Q262E also significantly reduced its binding to PGC-1α [37]. Coimmunoprecipitation and reporter assays indicated that all three mutants could still interact with TBK1 (Fig 6F) and inhibited TBK1-induced IFN-I activation (Fig 6D) to a similar extent as WT ERRα. ERRα negatively regulates TLR4 induced inflammation partially mediated by transcriptional activation of A20 [26]. To assess the role of A20 in ERRα-mediated antiviral signaling, we transfected Flag-ERRα into A20 knockout 293T cells generated by the CRISPR-Cas9 system (A20^CRISPR-/-^). We observed that the suppression abilities of ERRα on IFN-β activation and IRF3 phosphorylation in response to VSV infection were unchanged by A20 deletion (Fig 6G and 6H). Taken together, these data suggest that the negative effect of ERRα on innate immune signaling is independent of its transcriptional activity and its cofactor PCG-1α.

### ERRα is stabilized by viral infection

**Fig 7:**
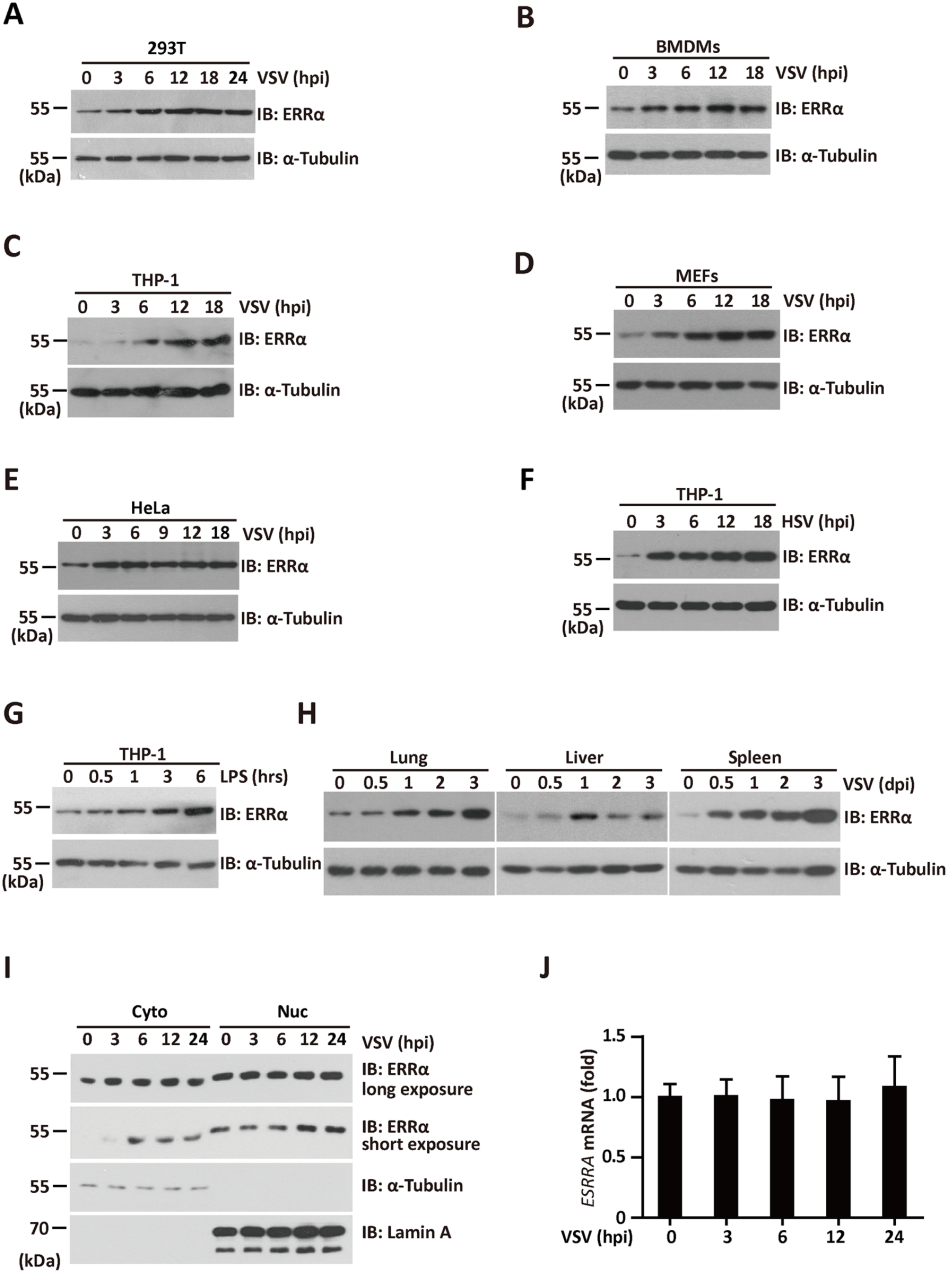
ERRα is stabilized by viral infection. (A-E) Immunoblotting analysis of ERRα protein expression in 293T (A), BMDMs (B), THP-1(C), MEFs (D), and HeLa (E) infected with VSV (MOI = 1.0) for the indicated times. α-Tubulin was used as the equal loading control. (F-G) Immunoblotting analysis of ERRα protein expression in THP-1 cells infected with HSV (F) or incubated with 100 ng/ml LPS (G) for the indicated times. α-Tubulin was used as the equal loading control. (H) Immunoblotting analysis of ERRα protein expression from tissues of VSV-infected C57BL/6 mice collected at the indicated time points (n = 3 per group). α-Tubulin was used as the equal loading control. (I) Immunoblotting analysis of fractionated 293T cells infected with VSV for the indicated times. Cyt, cytosolic; Nuc, nuclear. The purity of the fractions was assessed by blotting for Lamin A (nuclear protein) and α-Tubulin (cytosolic protein). (J) qRT-PCR analysis of ERRα mRNA expression in 293T cells infected with VSV (MOI = 1.0) for the indicated times. The data were normalized to the expression of the β-actin reference gene. Cell-based studies were performed independently at least three times with comparable results. The data are presented as the means ± SEM.

Because ERRα inhibited the production of interferons and regulated antiviral immunity, we examined whether ERRα was induced after viral infection. Protein levels of ERRα in 293T, BMDMs, THP-1, mouse embryonic fibroblasts (MEFs) and HeLa cells were increased significantly and rapidly following VSV infection (Fig 7A–7E). Moreover, ERRα expression was induced in THP-1 macrophages infected with HSV-1 (Fig 7F) or treated with LPS (Fig 7G). ERRα protein levels were also induced at 12 and 24 hpi in the lung, liver, and spleen of mice infected by tail vein injection of VSV, with the highest induction observed in the spleen (Fig 7H). Cytosolic redistribution of ERRα has been reported in response to HCMV infection [27]. Consistent with this result, nuclear-cytoplasmic fractionation of VSV-infected cells at different time points showed that ERRα was upregulated exclusively in the cytoplasm at 3 hpi, and this upregulation lasted until 24 hpi (Fig 7I). VSV infection also caused a mild upregulation of nuclear ERRα. Interestingly, nuclear ERRα migrated slower than the cytoplasmic form. The expression of ERRα mRNA did not change by VSV infection (Fig 7J). These results indicate that ERRα is stabilized by a post-transcriptional mechanism following viral infection.

### TBK1 is indispensable for viral-induced ERRα stabilization

**Fig 8:**
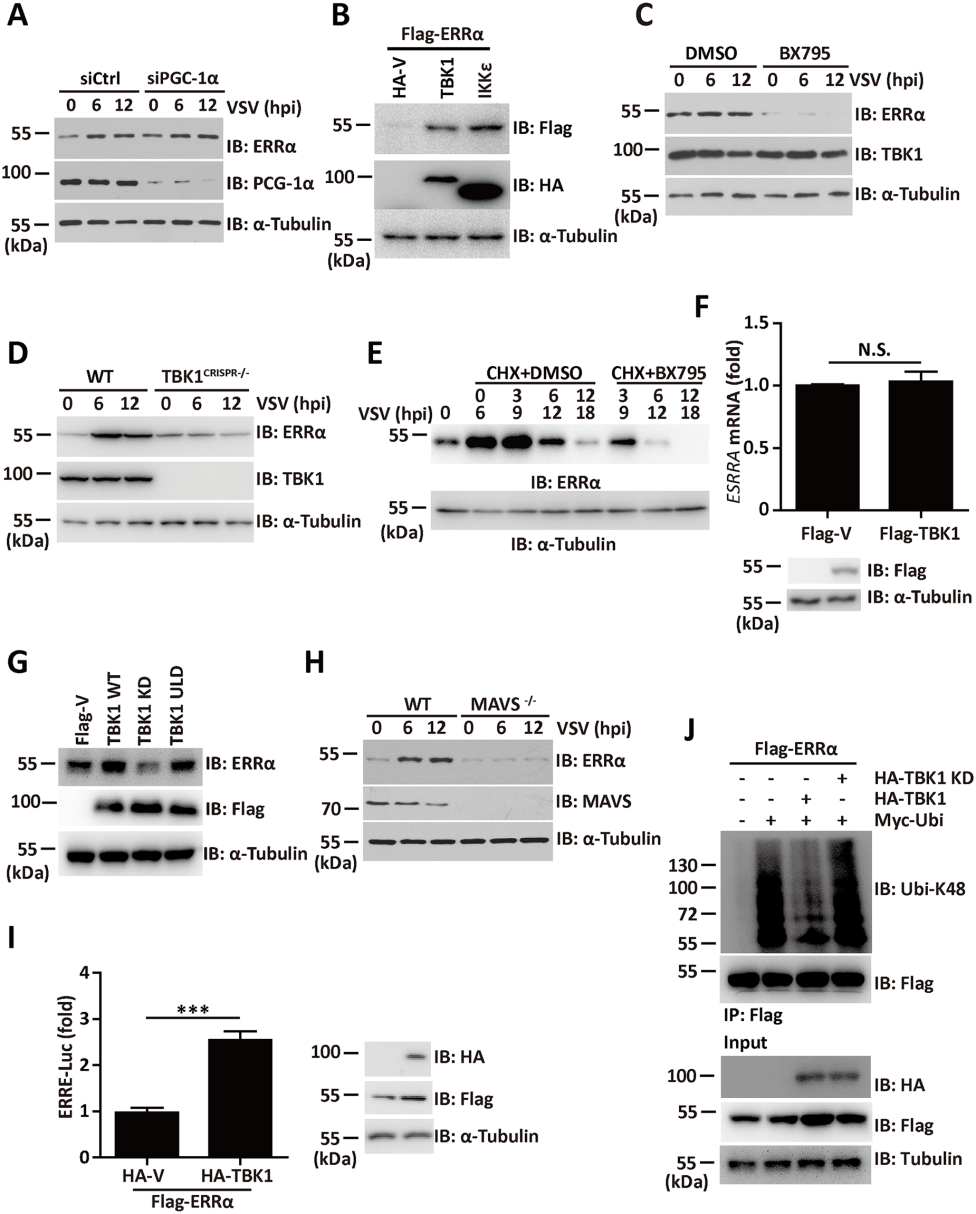
TBK1 is indispensable for viral-induced ERRα stabilization. (A) Immunoblotting analysis of ERRα protein expression in siCtrl or siPGC-1α cells infected with VSV (MOI = 1.0) for the indicated times. α-Tubulin was used as the equal loading control. (B) Immunoblotting analysis of ERRα protein expression in 293T cells transfected with Flag-ERRα and HA-TBK1 or HA-IKKε. α-Tubulin was used as the equal loading control. (C) Immunoblotting analysis of ERRα protein expression in 293T cells infected with VSV (MOI = 1.0) in the presence or absence of 1 μM BX795 for the indicated times. α-Tubulin was used as the equal loading control. (D) Immunoblotting analysis of ERRα protein expression in WT and TBK1^CRISPR-/-^ cells infected with VSV (MOI = 1.0) for the indicated times. α-Tubulin was used as the equal loading control. (E) Immunoblotting analysis of ERRα protein expression in 293T cells infected with VSV (MOI = 1.0) and treated with CHX in the presence or absence of 1 μM BX795 for the indicated times. (F) qRT-PCR analysis of ERRα mRNA expression in 293T transfected with Flag-TBK1. The data were normalized to the expression of the β-actin reference gene. (G) Immunoblotting analysis of ERRα protein expression in 293T cells transfected with the indicated plasmids. α-Tubulin was used as the equal loading control. (H) Immunoblotting analysis of ERRα protein expression in WT and MAVS-KO MEFs infected with VSV (MOI = 1.0) for the indicated times. (I) ERRα responsive promoter luciferase activity assay in 293T cells transfected with HA-TBK1 and Flag- ERRα. (J) Immunoprecipitation analysis of K48 linked-ubiquitination of ERRα in 293T cells transfected with Flag- ERRα, HA-TBK1 or HA-TBK1 KD together with Myc-ubiquitin in the presence of MG132. Loading controls were shown in the lower panel of some Figures. Cell-based studies were performed independently at least three times with comparable results. The data are presented as the means ± SEM.

Although the natural ligand of ERRα is unknown, ERRα can be activated by several cytokines and by PGC-1α. To determine the contribution of PGC-1α to viral-induced ERRα activation, we evaluated the contribution of PGC-1α to ERRα activation in response to viral infection. As shown in Fig 8A, PGC-1α knockdown cells also exhibited an induction of ERRα expression after VSV infection, indicating that other factors are involved in viral-induced ERRα stabilization. The association between ERRα and TBK1 prompted us to analyze the effect of TBK1 on ERRα expression in response to viral infection. When expression plasmids encoding TBK1 or IKKε were transfected into 293T cells with Flag-ERRα, a significant enhancement in the cellular abundance of ERRα was found (Fig 8B). Notably, the VSV-mediated expression of ERRα was completely inhibited by the TBK1 inhibitor BX795 (Fig 8C). In addition, we found that the induction of ERRα triggered by VSV infection was severely impaired in TBK1 defective cells (TBK1^CRISPR-/-^; Fig 8D). The half-life of ERRα was greatly reduced in the presence of BX795 (Fig 8E). QRT-PCR analysis showed no significant difference in the transcriptional level of ERRα following TBK1 overexpression (Fig 8F).

We next wanted to explore the role of TBK1 kinase activity on ERRα stabilization. Overexpression of WT TBK1, but not the TBK1 K38A kinase dead mutant (in which the ATP binding residue Lys38 was mutated to alanine), caused increased stabilization of ERRα (Fig 8G). ULD-mutated TBK1 (TBK1 L352A, I353A) which failed to activate the NF-κB, IFN-β and IRF3 promoter as shown previously [38], retained its ability to induce ERRα expression (Fig 8G). Therefore, TBK1 phosphorylation rather than the TBK1-mediated antiviral response is required for viral-mediated stabilization of ERRα. Consistent with the essential role of MAVS in TBK1 phosphorylation and activation, MAVS-KO MEFs failed to stabilize ERRα in response to VSV infection (Fig 8H). TBK1 overexpression also increased the expression of the ERRα target gene ERRE [39], as shown by the luciferase reporter assay (Fig 8I). These results indicated that TBK1 is required for viral induced ERRα stabilization.

To further delineate the mechanism of TBK1-mediated ERRα stabilization, Myc-ubiquitin was cotransfected with plasmids expressing Flag- ERRα together with HA-TBK1 or HA-TBK1 KD in the presence of proteasome inhibitors MG132, ERRα was then immunoprecipitated by anti-Flag antibody and blotted with anti-Ubi-K48 antibody. Immunoprecipitation assay showed that overexpression of WT TBK1, but not TBK1 KD mutant, led to a sharp reduction on the K48 ubiquitination level of ERRα (Fig 8J), suggesting that TBK1 phosphorylation modification might contribute to the stabilization of ERRα by inhibiting its K48-linked polyubiquitylation.

### ERRα chemical inhibitor XCT790 has broad antiviral potency

**Fig 9:**
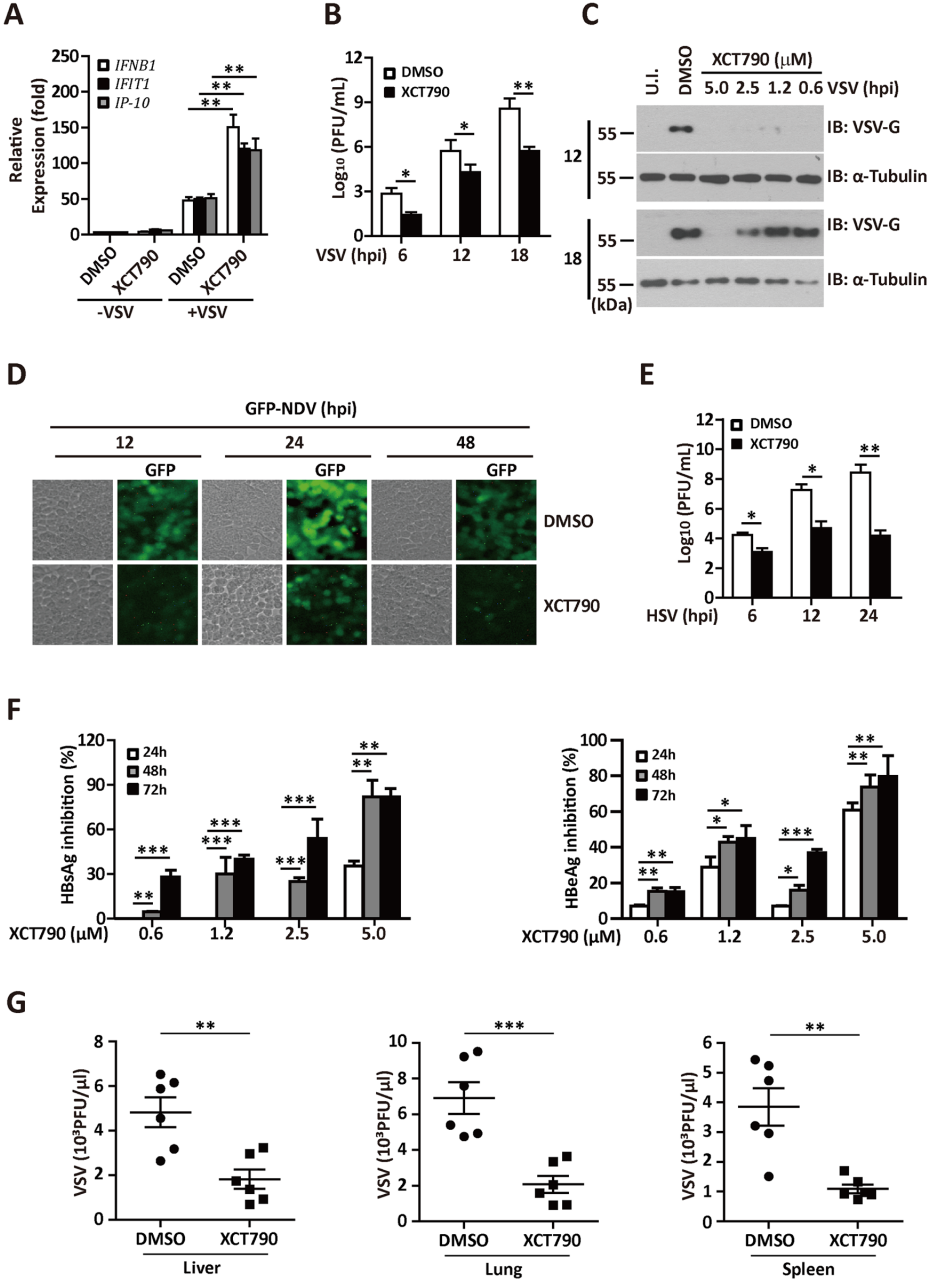
ERRα chemical inhibitor XCT790 has broad antiviral potency. (A) qRT-PCR analysis of *IFNB*, *IFIT1* and *IP-10* mRNA expression in 293T cells infected with VSV (MOI = 1.0) in the presence or absence of 2.5 μM XCT790 for the indicated times. The data were normalized to the expression of the β-Actin reference gene. (B) Plaque assay of VSV loads in supernatants of 293T cells infected with VSV (MOI = 1.0) in the presence or absence of 2.5 μM XCT790 for the indicated times. (C) Immunoblotting analysis of VSV-G protein expression in supernatants of 293T cells infected with VSV (MOI = 1.0) in the presence of the indicated dose of XCT790 for the indicated times. (D) Representative images in 293T cells infected with NDV-GFP in the presence or absence of XCT790 for the indicated times. (E) Plaque assay of HSV-1 titers in the supernatants of 293T cells in the presence or absence of 5 μM XCT790 for the indicated times. (F) ELISA assay of HBsAg (left panel) or HBeAg (right panel) in the supernatants of HepG2.2.15 cells treated with DMSO or XCT790 for the indicated times. (G) Viral titer analysis of VSV production in lung, liver or spleen isolated from VSV infected mice treated with DMSO or XCT790 (n = 6 per group). Cell-based studies were performed independently at least three times with comparable results. The data are presented as the means ± SEM.

Next, we investigated the antiviral activity of XCT790, a synthetic antagonist of ERRα. First, the effect of XCT790 on IFN-I induction was explored. As shown in Fig 9A, the mRNA expression levels of VSV-induced IFN-β and the IFN-regulated gene products *IFIT1* and *IP-10* were significantly upregulated in XCT790-treated cells. Accordingly, treatment of 293T cells with XCT790 inhibited VSV production (Fig 9B) and VSV-G expression (with IFN-β and 25-HC as the positive control) (S6A Fig). XCT790 inhibited VSV-G protein expression in a dose-dependent manner (Fig 9C). A similar antiviral effect was observed in several human cell lines, including HeLa, A549, and primary isolated BMDMs (S6B Fig). A cytoprotective effect of XCT790 in 293T, HeLa, and A549 cells upon VSV infection was also observed (S6C Fig). To determine the breadth of the antiviral activity of XCT790, we tested the effect of XCT790 on various viruses. By quantifying NDV-GFP using flow cytometry and fluorescence microscopy analysis, we found that XCT790 inhibited NDV-GFP replication in 293T cells by over 90% (Fig 9D and S6D Fig). Treatment of A549 cells (S6E Fig) with XCT790 inhibited NDV-GFP expression by approximately 50%. XCT790 also inhibited HSV-1 production in 293T cells (Fig 9E). Treatment with the indicated dose of XCT790 reduced HBV surface antigen (HBsAg) and e antigen (HBeAg) expression by 50% (Fig 9F).

To further evaluate the antiviral role of XCT790 *in vivo*, we intravenously administered VSV in mice treated with XCT790 or DMSO. As expected, the XCT790-treated mice had a much lower virus load in the serum, liver and lung (Fig 9G). Taken together, our results demonstrate that ERRα chemical inhibitor XCT790 exhibits antiviral activity against several types of viruses.

## Discussion

Innate immunity and metabolism appear to be inextricably linked and are now known to regulate each other reciprocally [16,40–42]. Exciting new evidence is emerging with regard to the role of TLRs and NLRs in the regulation of metabolism and the activation of inflammatory pathways during the progression of metabolic disorders, such as type 2 diabetes [43] and Reye's syndrome [44]. Studies have also suggested that metabolites, such as 25-HC [45–47], NAD [48] (acting via deacetylases such as SIRT1 and SIRT2) and succinate [49] (which regulates hypoxia-inducible factor 1), regulate innate immunity. Additionally, extracellular overproduction of metabolites, such as uric acid and cholesterol crystals, is sensed by NLRP3, leading to activation of the inflammasome complex and the production of IL-1β [50,51]. In turn, some nuclear receptors reported to regulate metabolism, such as the glucocorticoid receptor (GR) [52], peroxisome proliferator-activated receptor γ (PPAR-γ) [53] and retinoid X receptor α (RXRα) [54], have been implicated in type I interferon regulation. The interplay between immunity and metabolic changes is a growing field of research.

This study investigated an unappreciated relationship between the host IFN-I response and ERRα, a member of the nuclear receptor superfamily involved in the transcriptional control of energy homeostasis. Several lines of evidence support this argument: (1) Viral infection led to increased ERRα expression both *in vivo* and *in vitro*. Further study showed that TBK1 was indispensable for viral-induced ERRα stabilization. (2) Overexpression of ERRα resulted in potent inhibition of virus-triggered IRF3 activation and IFN-β induction, while inhibition of ERRα by knockdown, chemical antagonist and knockout enhanced IFN-β production and increased resistance to VSV, NDV, HSV and HBV infections. (3) Mechanistically, ERRα disrupted the TBK1-IRF3 interaction and the homo-dimerization of IRF3 by interacting with TBK1, IKKε and IRF3, which are critical for virus-induced IRF3 activation and IFN-β induction. (4) The effect of ERRα on IFN-I production was independent of its transcriptional activity and PCG-1α. Therefore, our findings indicate that ERRα serves as a negative regulator downstream of TBK1 that attenuates the persistence of the antiviral state independently of its role in metabolic signaling.

TBK1 is a key Ser-Thr kinase involved in innate immunity that is activated by adaptors, such as STING, TRIF and MAVS [55]. Activation of TBK1 leads to adaptor phosphorylation, IRF3 activation and expression of IRF3-dependent genes that are important for the response to viral infection; thus, their activities are tightly regulated. In addition to ERRα, MIP-T3 and SIKE have been identified as two other physiological suppressors that negatively regulate IFN-β production by inhibiting the formation of functional TBK1 complexes [11,12]. SIKE and ERRα disrupted the TBK1-IRF3 association by targeting TBK1, while MIP-T3 disrupted the TRAF3-TBK1 association through its direct interaction with TRAF3. Although both SIKE and ERRα are associated with TBK1 under physiological conditions, only the ERRα protein level was significantly increased in response to VSV infection. Hence, our work indicated that TBK1 activation not only activated IRF3 but also activated ERRα to affect both IFN-I induction and metabolic signaling, indicating the unique status of ERRα among the cellular inhibitors of innate immunity. Hwang reported that ERRα provides a metabolic environment to promote the production of cytomegalovirus [27]. In our study, we also showed that ERRα upregulation can be detected as early as 3 hpi. We speculated that the upregulation of ERRα at the early stage of viral infection may present a general strategy by which the host produces the energy to counteract the stress; however, the pathogen hijacks the host cell metabolic environment. Thus, pharmacological targeting of ERRα to uncouple pathogens from their nutritional dependencies and the host negative innate immune response may prove to be an effective strategy for controlling pathogen spread.

ERRα functions downstream of PGC-1α and PGC-1β and controls the expression of genes involved in metabolism. The upregulation of ERRα and the unchanged expression of PGC-1α in response to viral infection imply that additional factors may be involved in the regulation of viral-induced ERRα upregulation. Here, we showed that ERRα was specifically stabilized in response to the virus infection downstream of TBK1. Sequence profiles of ERRα across mammalian species revealed several putative consensus TBK1 phosphorylation motifs. We speculated that ERRα might be phosphorylated and activated by TBK1. In support of this hypothesis, TBK1 kinase activity was required for ERRα activation. Recent reports have shown that MAVS phosphorylated by TBK1 relays its downstream signal to IRF3 for its phosphorylation and activation by TBK1. Interestingly, MAVS knockout cells displayed both defective basal and activated ERRα in response to VSV infection. However, the phosphorylation of ERRα by TBK1 and the roles of adaptors in TBK1-mediated ERRα activation require further investigation.

In a recent work, TBK1 was shown to be highly expressed in lung, breast and colon cancers, and subjects with tumors that highly express TBK1 have poor responsiveness to tamoxifen treatment and a high potential for relapse [35]. ERRα expression was markedly increased in neoplastic versus normal tissues, and ERRα-positive tumors were associated with more invasive disease and a higher risk of recurrence [56]. We established that ERRα associates directly with TBK1; thus, ERRα might affect cancer progression as a substrate of the TBK1 kinase in addition to cooperating with TBK1 in the regulation of innate immune signaling. Indeed, breast cancer patients with hypo-phosphorylated ERRα are more likely to respond to hormonal-blockade therapy and have a longer overall survival time than those with hyper-phosphorylated ERRα, which may be a direct consequence of TBK1-mediated ERRα phosphorylation [57]. Our studies also suggest the possibility that viral infection induced ERRα activation may be a tumor-promoting factor, especially in persistent infection, but further investigation is required. These findings influence our understanding of the complex relationship between innate immune effectors, metabolic regulators and the signaling events that drive tumor formation.

Here, we provided direct evidence indicating the critical role of ERRα in virus replication by modulating IFN-I induction independent of its transcriptional activity. In line with this finding, the inhibition of ERRα effectively reduced the yield of VSV, NDV, HSV and HBV and showed a promising cytoprotective effect in response to viral infection in multiple cell lines. As ERRα is a potential target for the treatment of breast cancer and metabolic disorders, several selective ligands against ERRα are being developed. Our studies thus suggest the potential new application of ERRα antagonists in the treatment of viral infection.

## Materials and methods

### Mice and ethics statement

ERRα-KO mice on a C57BL/6J background were purchased from the Jackson Laboratory and maintained in specific pathogen–free conditions. All animals were handled in strict accordance with the Guide for the Care and Use of Laboratory Animals and the principles for the utilization and care of vertebrate animals, and all animal work was approved by the Institutional Animal Care Committee of Beijing Institute of Biotechnology. Animal experiments were performed in accordance with the regulations in the Guide for the Care and Use of Laboratory Animals published by the Ministry of Science and echnology of the People’s Republic of China. The protocol was approved by the ethics committee of Beijing Institute of Biotechnology (Permit Number: 2008–09).

### Plasmids

Mammalian expression plasmids pCMV-Flag-ERRα and HA-STING were provided by Dr. Toren Finkel [58] and Hongbin Shu [59]. Expression plasmids for pEBB-HA-TBK1 and pEBB-HA-IKKε were gifts from Dr. Genhong Cheng [60]. Gal4-Luc and Gal4-IRF3 were obtained from Zhijian J. Chen [34]. The ERRE luciferase plasmid was a gift from Timothy F. Osborne. IRF3 and RIG-I cDNA were amplified from a human spleen library and subsequently cloned into CMV promoter-based vectors. Other tagged cDNA containing plasmids and mutants were constructed by PCR amplification based on these plasmids. IFN-β-Luciferase and ISRE-Luciferase reporter plasmids were purchased from Beyotime Corp. Other mammalian expression vectors encoding Flag-, Myc-, or HA-fusion proteins tagged at the amino terminus were constructed by inserting PCR-amplified fragments into pcDNA3 (Invitrogen) or pCMV (Clontech). Plasmids encoding GST fusion proteins were generated by cloning PCR-amplified sequences into pGEX4T-1 (Amersham Pharmacia Biotech). HuSH 29mer shRNA constructs against ERRα kit was purchased from OriGene Company. The sequence of effective shRNA that targeted ERRα is GCAAAGCCTTCTTCAAGAGGACCATCCAG. A non-effective 29-mer scrambled shRNA cassette in the same vector from the kit was used as a negative control. All plasmids were verified by restriction enzyme analysis and DNA sequencing.

### Microarray

Total RNA from the cells with or without virus infection was quantified by the NanoDrop ND-2000 spectrophotometer (Thermo Scientific), and the RNA integrity was assessed using the Agilent Bioanalyzer 2100 (Agilent Technologies). The sample labeling, microarray hybridization and washing were performed based on the manufacturer’s standard protocols. Briefly, total RNA were transcribed to double stranded cDNA, then synthesized into cRNA and labeled with Cyanine-3-CTP. The labeled cRNAs were hybridized onto the Agilent Human Gene Expression (8*60K, Design ID: 039494) microarray. After washing, the arrays were scanned by the Agilent Scanner G2505C (Agilent Technologies). Feature Extraction software (version 10.7.1.1, Agilent Technologies) was used to analyze array images to obtain raw data. Genespring was employed to complete the basic analysis of the raw data. First, the raw data were normalized with the quantile algorithm. Then, GO analysis and KEGG analysis were applied to determine the roles of these differentially expressed mRNAs.

### BMDM isolation

BMDMs were isolated from WT and ERRα-KO C57BL/6 mice by culturing for 6 days in RPMI 1640 medium containing 10 ng/ml M-CSF (PeproTech). Twenty-four hours prior to infection, 1 x 10^6^ cells were seeded into 12-well plates with RPMI 1640 containing 10 ng/ml M-CSF and 10% fetal bovine serum (FBS, HyClone).

### Cell culture and transfection

Human cell lines 293T, HeLa, A549, mouse embryonic fibroblasts (MEFs) and HepG2.2.15 were routinely cultured in DMEM (Invitrogen) containing 10% FBS (HyClone). 293T, HeLa and A549 cell lines were obtained from ATCC. MEFs and HepG2.2.15 cells were gifted from Dr. Cheng Cao. Cells were maintained as monolayers in a humidified atmosphere containing 5% CO_2_ at 37°C. Lipofectamine 2000 reagent was used for transfection following the manufacturer’s protocol (Invitrogen). Stable cell lines were selected in 1 μg/ml puromycin for approximately 2 weeks. Individual clones were screened by standard immunoblotting protocols and produced similar results. The luciferase reporter assay was performed as described previously [61].

### VSV, HSV-1 and NDV infection

VSV and NDV were kindly provided by Dr. Cheng Cao. HSV-1 was donated by Dr. Wei Chen. Cells were infected with the virus at the indicted MOI for 1 h, and then the media was replaced with fresh media. For HSV-1 and VSV, supernatants were collected, and titers were measured by plaque assays using BHK21 cells [54,62].

### Western blotting and immunoprecipitation

Cell extracts were prepared, immunoprecipitated and analyzed as previously described [63]. An aliquot of the total lysate (5%, v/v) was included as a control for the interaction assay. Immunoprecipitation was performed with an anti-Flag M2 Affinity Gel (Sigma-Aldrich, A2220) and anti-ERRα (Epitomics, 2131–1). Western blotting was performed by HRP-labeled anti-Myc (Sigma-Aldrich, A5598), anti-HA (Sigma-Aldrich, H9658), anti-TBK1 (Epitomics, 3296–1), anti-ERRα (Epitomics, 2131–1), anti-IRF3 (pSer386) (Epitomics, 2346), anti-IRF3 (pSer396) (Cell Signaling Technology, 4947s), anti-A20 (ABclonal, A2127), anti-IKKε (ABclonal, A0244) or anti-α-Tubulin (Sigma-Aldrich, T6074) antibodies. The antigen-antibody complexes were visualized by chemiluminescence.

In a Far-Western assay, immunoprecipitates were separated by SDS-PAGE and then blotted onto nitrocellulose membranes. The membranes were subsequently incubated with purified GST-fusion proteins for 1 h at room temperature. The GST fusion proteins binding to nitrocellulose were probed with an anti-GST antibody.

### Quantitative real-time RT-PCR (qRT-PCR)

qRT-PCR was performed in the iQ5 Real-time PCR System (Bio-Rad) using iTaq universal SYBR Green supermix (Bio-Rad). Each sample was analyzed in triplicate with GAPDH as the internal control. S1 Table lists the primer sequences used for different genes in this study.

### Native gel electrophoresis

Cells were lysed in NP-40 lysis buffer as previously described [65] and mixed with native loading buffer (250 mM Tris-HCl (pH 7.5), 50% glycerol and 0.007% xylene cyanol). The 8% native gel was pre-run with 25 mM Tris and 192 mM glycine with 1% deoxycholate (DOC) in the inner chamber for 30 min at 40 mA. Then, the samples were resolved for 60 min at 40 mA at 4°C [64]. The proteins from the native gel were transferred to PVDF membranes for immunoblotting analysis, as described above.

### In situ PLA

Fixed and permeabilized cells were incubated overnight at 4°C with the following pairs of primary antibodies: anti-ERRα (Epitomics, 2131–1), mouse mAb to IRF3 (BioLegend, 655701) or mouse mAb to TBK1 (Santa Cruz, sc-398366). The cells were washed and allowed to react with a pair of proximity probes (Olink Bioscience). The remainder of the *in situ* PLA protocol was performed according to the manufacturer’s instructions. The cells were examined by fluorescence microscopy (UlthaView VOX, PerkinElmer), and the Duolink Image Tool (Olink Bioscience) was used for quantitative analysis.

### Luciferase reporter assays

293T cells cultured in 24-well plates were transfected using Lipofectamine 2000 with 0.1 μg of reporter, 0.002 μg of the pRL control vector, and various amounts of the indicated constructs. After incubation for 24 h, the cells were harvested, and luciferase activity was analyzed using the Dual Luciferase Reporter Assay System (Promega). Total light production was measured with a TD-20/20 Single-Tube Luminometer (Turner BioSystems). All experiments were repeated at least three times.

### Measurement of cytokines

Human and mouse IFN-β were quantified with IFN-β ELISA kits from Antigenix (4756) and Biolegend (3861), respectively.

### Histology

The lungs from control or virus-infected mice were washed with PBS, and then fixed in 4% PBS-buffered paraformaldehyde for 12 h, embedded into paraffin, sectioned, stained with hematoxylin and eosin solution.

### ChIP assay

293T control cells or ERRα knockdown cell lines were infected by VSV for the indicated time and subjected to the ChIP assay using anti-IRF3 or control mouse IgG. The IFN-β enhancer region was amplified by PCR using specific primers as follows [65]: Sense, 5′-GAATCCACGGATACAGAACCT-3′, Antisense, 5′-TTGACAACA-CGAACAGTGTCG-3′. Amplification of the total input DNA was shown as an equal loading control. The experiment was performed as described in reference [65].

### Statistical analysis

Significant differences were calculated using a paired Student’s *t*-test. *p < 0.05, **p < 0.01 and ***p < 0.001. Estimation of overall survival was performed using Kaplan–Meier analysis, and differences between curves were compared using log-rank tests.

## Supporting information

S1 Fig ERRα deficiency confers resistance to viral infection *in vitro*, related to Fig 1. (A) Immunoblotting analysis of ERRα expression in WT and ERRα-KO BMDMs; α-Tubulin was used as the equal loading control. (B) Immunoblotting analysis of ERRα expression in control (shCtrl) and two stable ERRα knockdown (shERRα-1 and shERRα-2) 293T cells. α-Tubulin was used as the equal loading control. (C) Cell viability analysis of shCtrl or shERRα-2 293T cells infected with VSV at the indicated MOI for 36 h. (D) Plaque assay of VSV loads in supernatants of 293T cells transfected with Flag-ERRα or Flag-vector (Flag-V), followed by VSV (MOI = 1.0) infection for the indicated times. (E) Immunoblotting analysis of ERRα expression in shCtrl and shERRα-2 293T cells; α-Tubulin was used as the equal loading control. (F) Flow cytometry analysis of NDV-GFP replication in shCtrl and shERRα-2 293T cells infected with NDV-GFP for 16 h. NDV-GFP replication was defined as the product of the percentage of GFP-positive cells and the geometric mean of the fluorescence intensity. Loading controls were shown in the lower panel of some Figures. Cell-based studies were performed independently at least three times with comparable results. The data are presented as the means ± SEM.
(TIF)

S2 Fig ERRα knockdown increases the expression of multiple antiviral genes, related to Fig 2. (A) Immunoblotting analysis of ERRα expression in shCtrl and shERRα-2 293T cells; α-Tubulin was used as the equal loading control. (B) Heatmap of downregulated metabolic genes.
(TIF)

S3 Fig ERRα negatively regulates type I interferon signaling pathways, related to Fig 3. (A) IFN-β promoter luciferase activity assays in siCtrl and siERRα A549 cells infected with VSV (MOI = 1.0) for 12 h (upper panel). Immunoblotting analysis of ERRα expression (lower panel). Loading controls were shown in the lower panel of some Figures. Cell-based studies were performed independently at least three times with comparable results. The data are presented as the means ± SEM.
(TIF)

S4 Fig ERRα associates with TBK1, IKKε and IRF3, related to Fig 4. (A) Immunoblotting analysis in 293T cells transfected with the indicated plasmids. (B-C) IRF3 (B) or ISRE (C) promoter luciferase activity assay in 293T cells transfected with the indicated plasmids. (D) Immunoblotting analysis of Flag-IRF3 and Myc-ERRα expression in supernatants of 293T cells transfected with the indicated plasmids; α-Tubulin was used as the equal loading control. (E) ISRE promoter luciferase activity assays in 293T cells transfected with the indicated plasmids. (F) Immunoblotting analysis of ERRα, Flag-TBK1 or Flag-IKKε expression in shCtrl and shERRα-2 293T cells; α-Tubulin was used as the equal loading control. Loading controls were shown in the lower panel of some Figures. Cell-based studies were performed independently at least three times with comparable results. The data are presented as the means ± SEM.
(TIF)

S5 Fig ERRα regulates TBK1-IRF3 complex formation, related to Fig 5. (A) Immunoblotting analysis of ERRα protein expression in supernatants of 293T cells treated with DMSO or 5 μM XCT790 for 9 h. Quantification of PLA signals per cell in Fig 5B presented relative to control cells treated with solvent. (C) Immunoprecipitation analysis in WT and ERRα^CRISPR-/-^ cells infected with VSV (MOI = 1.0) for the indicated times. (D) IFN-β promoter luciferase activity assay in 293T cells transfected with Flag-ERRα or its truncated mutants and infected with VSV (MOI = 1) for 6 h. (E) IFN-β promoter luciferase activity assay in 293T cells transfected with Flag-ERRα or its truncated mutants together with HA-TBK1. (F) ISRE luciferase activity an assay in 293T cells transfected with Flag-ERRα or its truncated mutants together with HA-TBK1. (G) Immunoblotting analysis of Flag-ERRα or its deletion mutants protein expression in Fig 5H. (H) Immunoblotting analysis of Flag-ERRα or its deletion mutants protein expression in Fig 5I. Loading controls were shown in the lower panel of some Figures. Cell-based studies were performed independently at least three times with comparable results. The data are presented as the means ± SEM.
(TIF)

S6 Fig ERRα regulates TBK1-IRF3 complex formation, related to Fig 9. (A) Immunoblotting analysis of VSV-G protein expression in supernatants of 293T cells infected with VSV for the indicated times with or without DMSO, XCT790, IFN-β or 25HC. (B) Immunoblotting analysis of VSV-G protein expression in supernatants of HeLa, A549 and BMDMs cells infected with VSV (MOI = 1.0) in the presence or absence of 5 μM XCT790 for the indicated times. (C) MTT assay to measure cell viability of 293T, HeLa, and A549 cells infected with VSV (MOI = 1.0) in the presence or absence of 5 μM XCT790 for the indicated times. (D) Flow cytometry analysis of NDV-GFP replication in 293T cells in the presence or absence of 5 μM XCT790 for the indicated times. (E) Representative images in A549 cells infected with NDV-GFP in the presence or absence of 5 μM XCT790 for the indicated times. Loading controls were shown in the lower panel of some Figures. The data are presented as the means ± SEM.
(TIF)

S1 Table Listing of primer sequences used in this study. (DOCX)
