# Supplementary figures and images for: ERRα negatively regulates type I interferon induction by inhibiting TBK1-IRF3 interaction
Source: PLoS Pathog. 2017 Jun 7;13(6):e1006347. doi: 10.1371/journal.ppat.1006347 (PMC5476288; doi:10.1371/journal.ppat.1006347)

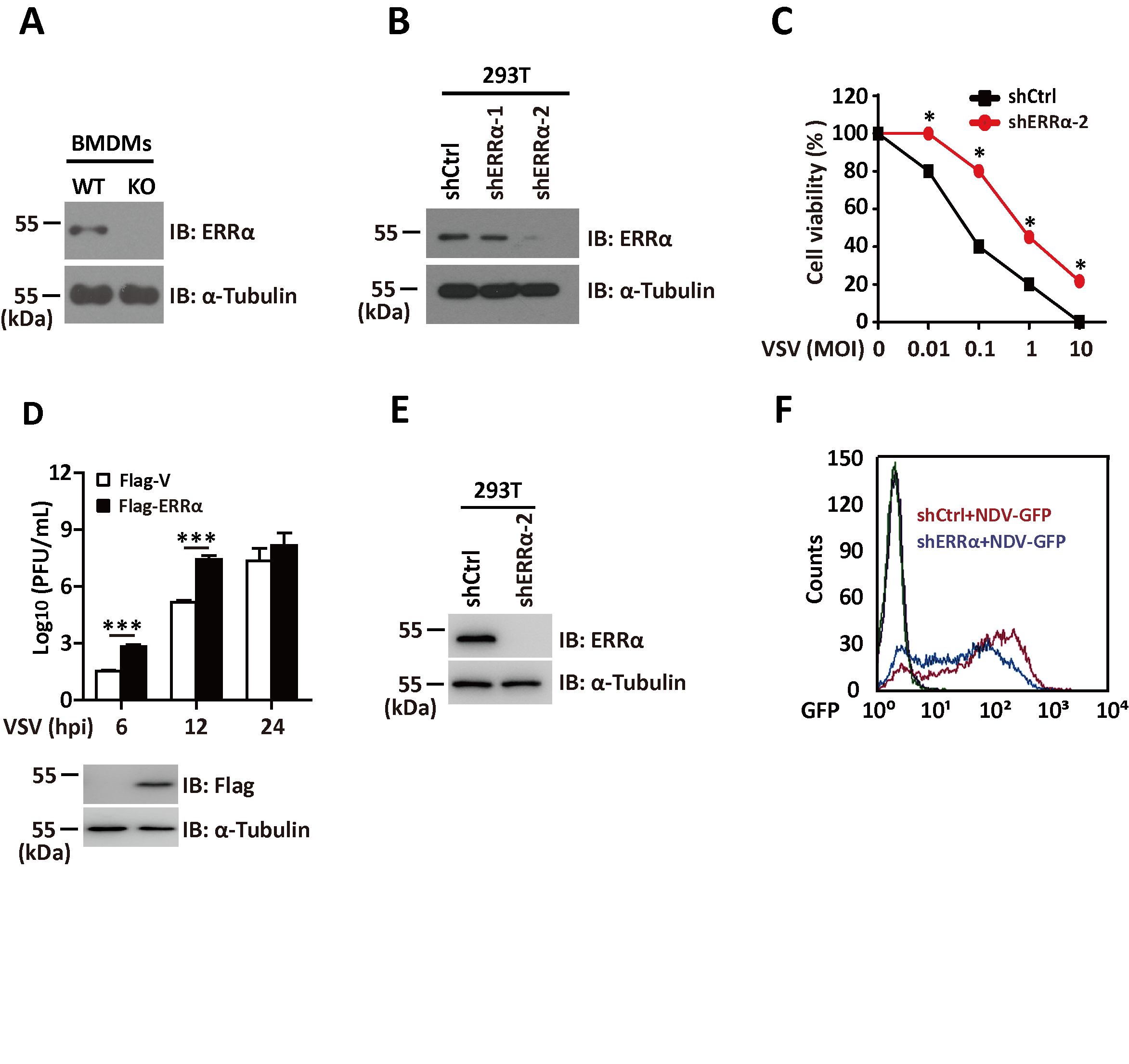

Supplement: S1 Fig — (A) Immunoblotting analysis of ERRα expression in WT and ERRα-KO BMDMs; α-Tubulin was used as the equal loading control. (B) Immunoblotting analysis of ERRα expression in control (shCtrl) and two stable ERRα knockdown (shERRα-1 and shERRα-2) 293T cells. α-Tubulin was used as the equal loading control. (C) Cell viability analysis of shCtrl or shERRα-2 293T cells infected with VSV at the indicated MOI for 36 h. (D) Plaque assay of VSV loads in supernatants of 293T cells transfected with Flag-ERRα or Flag-vector (Flag-V), followed by VSV (MOI = 1.0) infection for the indicated times. (E) Immunoblotting analysis of ERRα expression in shCtrl and shERRα-2 293T cells; α-Tubulin was used as the equal loading control. (F) Flow cytometry analysis of NDV-GFP replication in shCtrl and shERRα-2 293T cells infected with NDV-GFP for 16 h. NDV-GFP replication was defined as the product of the percentage of GFP-positive cells and the geometric mean of the fluorescence intensity. Loading controls were shown in the lower panel of some Figures. Cell-based studies were performed independently at least three times with comparable results. The data are presented as the means ± SEM. (TIF) [file ppat.1006347.s001.tif]

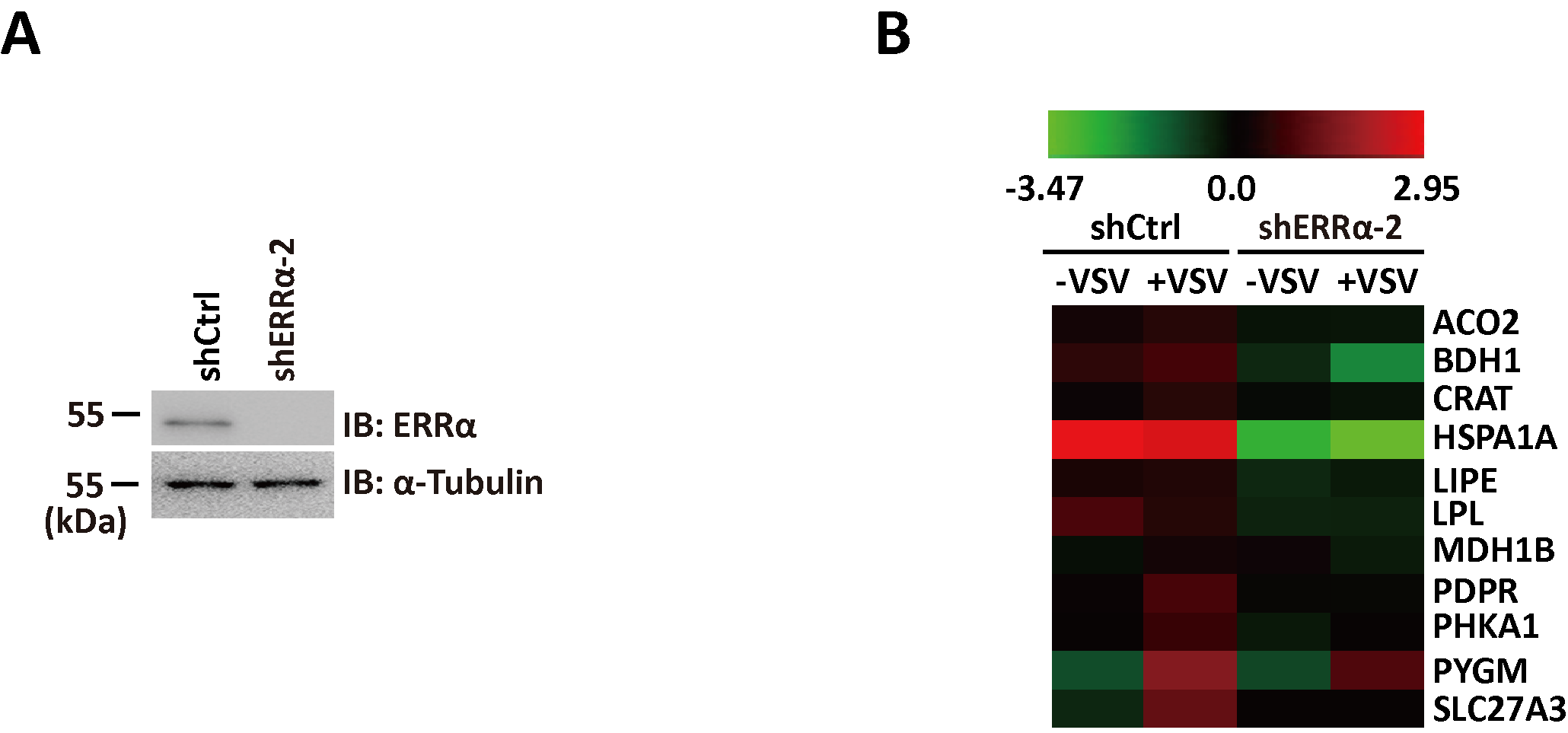

Supplement: S2 Fig — (A) Immunoblotting analysis of ERRα expression in shCtrl and shERRα-2 293T cells; α-Tubulin was used as the equal loading control. (B) Heatmap of downregulated metabolic genes. (TIF) [file ppat.1006347.s002.tif]

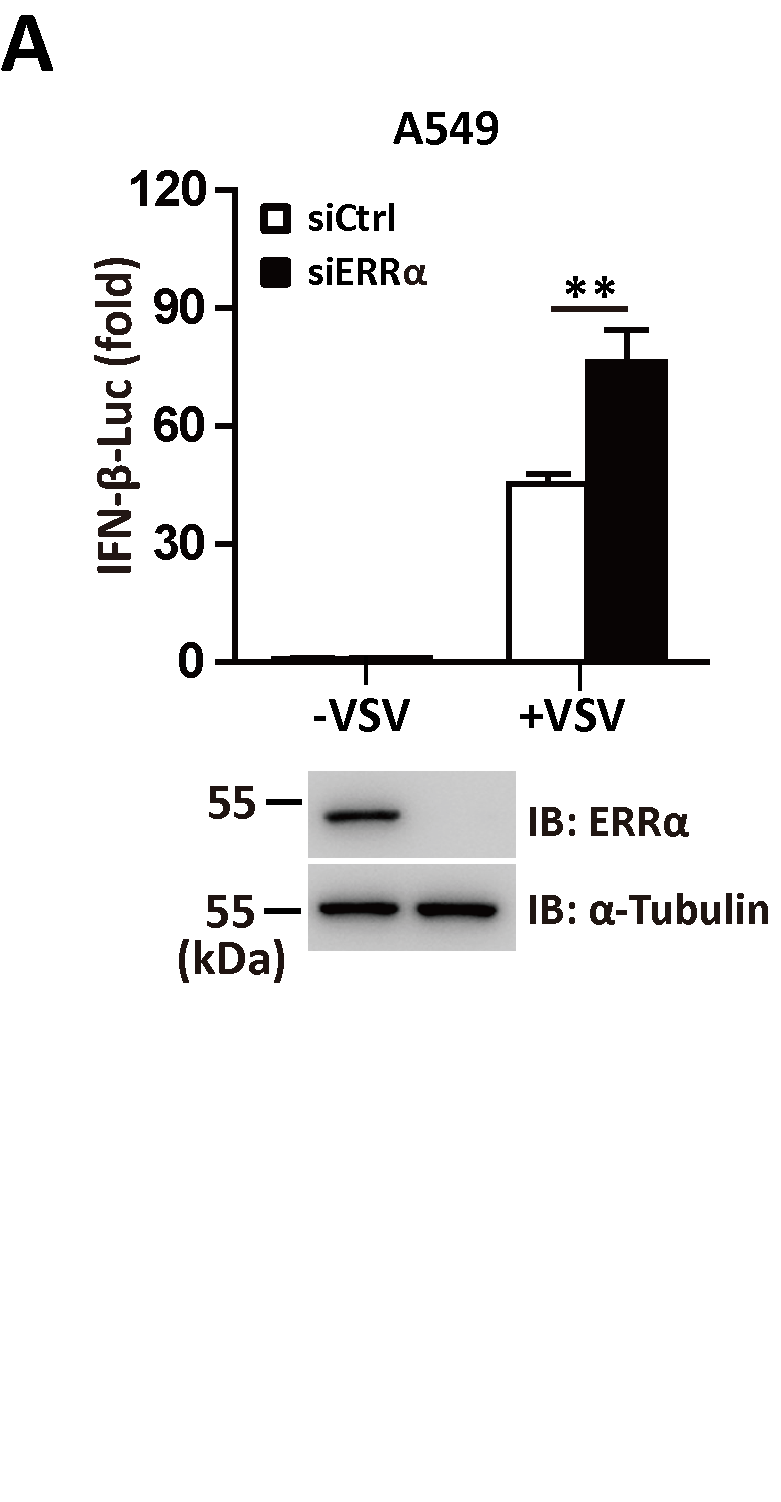

Supplement: S3 Fig — (A) IFN-β promoter luciferase activity assays in siCtrl and siERRα A549 cells infected with VSV (MOI = 1.0) for 12 h (upper panel). Immunoblotting analysis of ERRα expression (lower panel). Loading controls were shown in the lower panel of some Figures. Cell-based studies were performed independently at least three times with comparable results. The data are presented as the means ± SEM. (TIF) [file ppat.1006347.s003.tif]

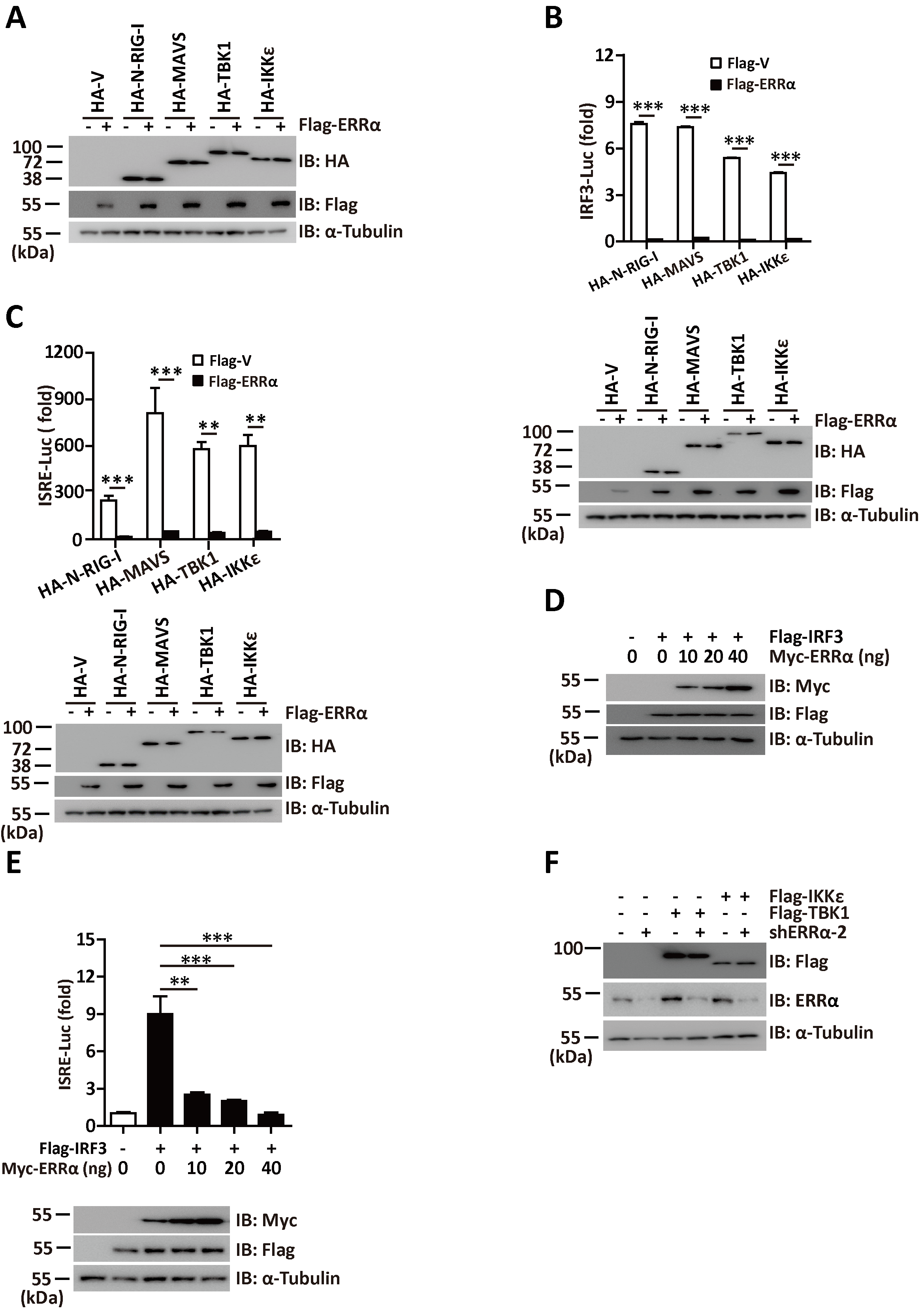

Supplement: S4 Fig — (A) Immunoblotting analysis in 293T cells transfected with the indicated plasmids. (B-C) IRF3 (B) or ISRE (C) promoter luciferase activity assay in 293T cells transfected with the indicated plasmids. (D) Immunoblotting analysis of Flag-IRF3 and Myc-ERRα expression in supernatants of 293T cells transfected with the indicated plasmids; α-Tubulin was used as the equal loading control. (E) ISRE promoter luciferase activity assays in 293T cells transfected with the indicated plasmids. (F) Immunoblotting analysis of ERRα, Flag-TBK1 or Flag-IKKε expression in shCtrl and shERRα-2 293T cells; α-Tubulin was used as the equal loading control. Loading controls were shown in the lower panel of some Figures. Cell-based studies were performed independently at least three times with comparable results. The data are presented as the means ± SEM. (TIF) [file ppat.1006347.s004.tif]

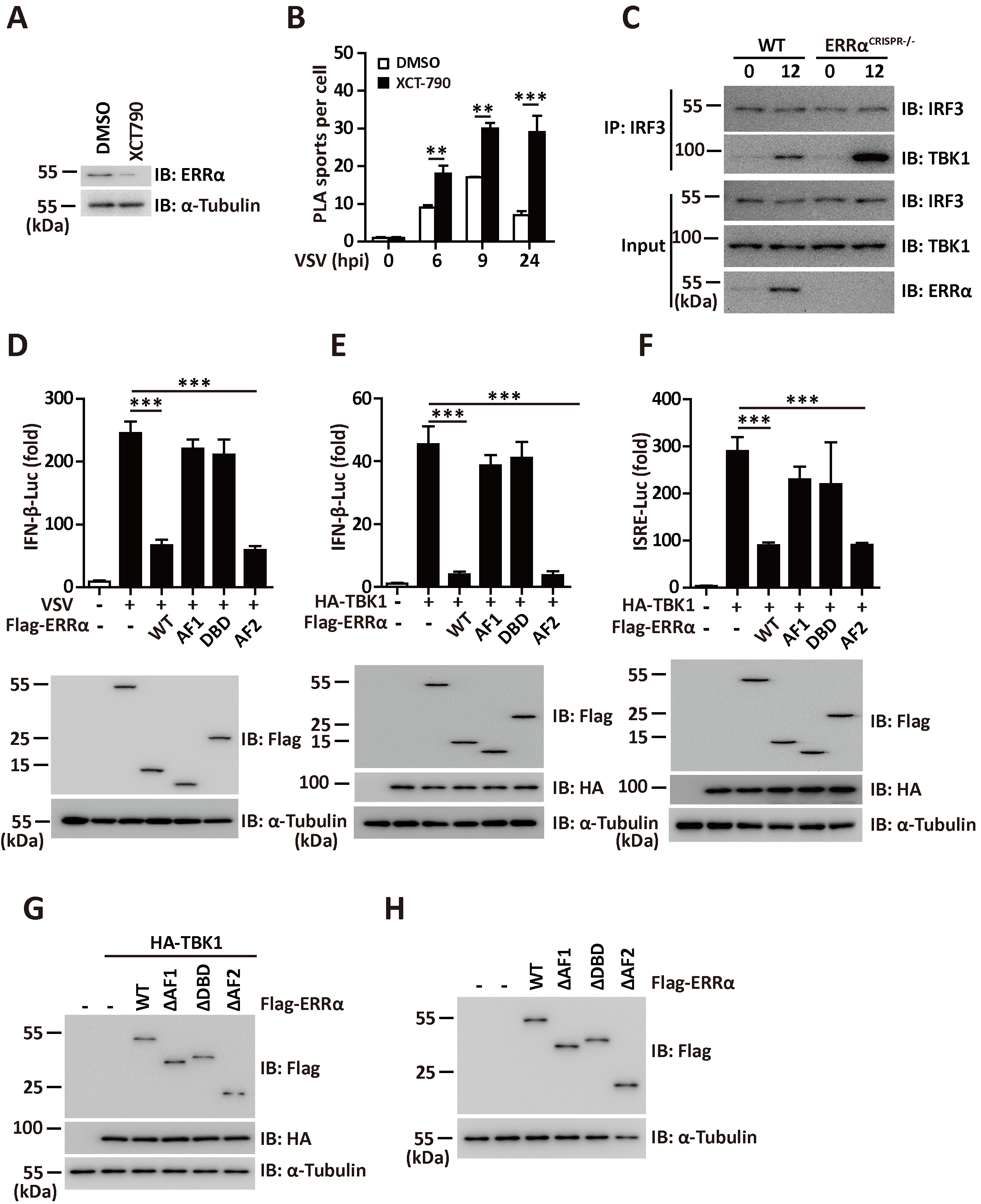

Supplement: S5 Fig — (A) Immunoblotting analysis of ERRα protein expression in supernatants of 293T cells treated with DMSO or 5 μM XCT790 for 9 h. Quantification of PLA signals per cell in Fig 5B presented relative to control cells treated with solvent. (C) Immunoprecipitation analysis in WT and ERRαCRISPR-/- cells infected with VSV (MOI = 1.0) for the indicated times. (D) IFN-β promoter luciferase activity assay in 293T cells transfected with Flag-ERRα or its truncated mutants and infected with VSV (MOI = 1) for 6 h. (E) IFN-β promoter luciferase activity assay in 293T cells transfected with Flag-ERRα or its truncated mutants together with HA-TBK1. (F) ISRE luciferase activity an assay in 293T cells transfected with Flag-ERRα or its truncated mutants together with HA-TBK1. (G) Immunoblotting analysis of Flag-ERRα or its deletion mutants protein expression in Fig 5H. (H) Immunoblotting analysis of Flag-ERRα or its deletion mutants protein expression in Fig 5I. Loading controls were shown in the lower panel of some Figures. Cell-based studies were performed independently at least three times with comparable results. The data are presented as the means ± SEM. (TIF) [file ppat.1006347.s005.tif]

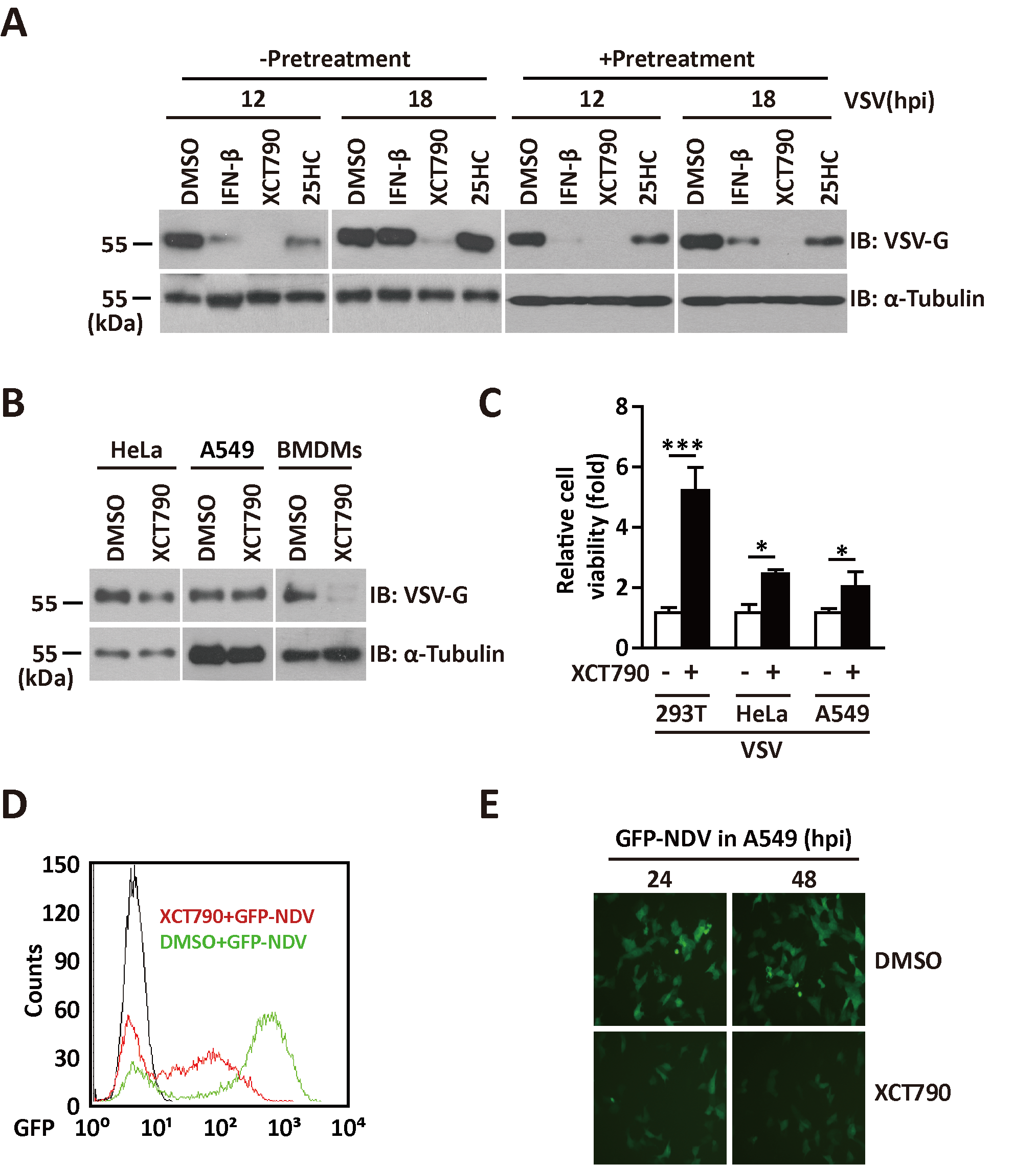

Supplement: S6 Fig — (A) Immunoblotting analysis of VSV-G protein expression in supernatants of 293T cells infected with VSV for the indicated times with or without DMSO, XCT790, IFN-β or 25HC. (B) Immunoblotting analysis of VSV-G protein expression in supernatants of HeLa, A549 and BMDMs cells infected with VSV (MOI = 1.0) in the presence or absence of 5 μM XCT790 for the indicated times. (C) MTT assay to measure cell viability of 293T, HeLa, and A549 cells infected with VSV (MOI = 1.0) in the presence or absence of 5 μM XCT790 for the indicated times. (D) Flow cytometry analysis of NDV-GFP replication in 293T cells in the presence or absence of 5 μM XCT790 for the indicated times. (E) Representative images in A549 cells infected with NDV-GFP in the presence or absence of 5 μM XCT790 for the indicated times. Loading controls were shown in the lower panel of some Figures. The data are presented as the means ± SEM. (TIF) [file ppat.1006347.s006.tif]
