# Supplementary material for: ERRα negatively regulates type I interferon induction by inhibiting TBK1-IRF3 interaction
Source: PLoS Pathog. 2017 Jun 7;13(6):e1006347. doi: 10.1371/journal.ppat.1006347 (PMC5476288; doi:10.1371/journal.ppat.1006347)
Supplement: S1 Table — (DOCX) [file ppat.1006347.s007.docx]

**Supplementary Table 1: List of Real time-PCR primers used in this study**

| **Name** | **Sequence (5’-3’)** |
| --- | --- |
| Human *ERRα* | Forward: CAAGAGCATCCCAGGCTT  Reverse: GCACTTCCATCCACACACTC |
| Human *IFN-β* | Forward: GTCAGAGTGGAAATCCTAAG  Reverse: ACAGCATCTGCTGGTTGAAG |
| Human *IFIT1* | Forward: TCGGAGAAAGGCATTAGATC  Reverse: GACCTTGTCTCACAGAGTTC |
| Human *IFIT2* | Forward: GACACGGTTAAAGTGTGGAGG  Reverse:TCCAGACGGTAGCTTGCTATT |
| Human *IFIT3* | Forward:TCAGAAGTCTAGTCACTTGGGG  Reverse:ACACCTTCGCCCTTTCATTTC |
| Human *IFIH1* | Forward:TCGAATGGGTATTCCACAGACG  Reverse:GTGGCGACTGTCCTCTGAA |
| Human *LILRB2* | Forward: GCATCTTGGATTACACGGATACG  Reverse: CTGACAGCCATATCGCCCTG |
| Human *β-Actin* | Forward: AAGGAGCCCCACGAGAAAAAT  Reverse: ACCGAACTTGCATTGATTCCAG |
| Mouse *IFN-β* | Forward:CAGCTCCAAGAAAGGACGAAC  Reverse:GGCAGTGTAACTCTTCTGCAT |
| Mouse *IFIT1* | Forward:CTGAGATGTCACTTCACATGGAA  Reverse:GTGCATCCCCAATGGGTTCT |
| Mouse *IP-10* | Forward: TCCCATCACTTCCCTACATG  Reverse: TGAAGCAGGGTCAGAACATC |
| Mouse *β-Actin* | Forward: ATGACCCAAGCCGAGAAGG  Reverse: CGGCCAAGTCTTAGAGTTGTTG |
